# Attention to facial emotions in adult women varies by type and severity of childhood maltreatment experience and emotion regulation strategy

**DOI:** 10.1038/s41598-025-99562-z

**Published:** 2025-05-09

**Authors:** Dennis Hoepfel, Anastasiia Bila, Vivien Günther, Anette Kersting, Thomas Suslow

**Affiliations:** Department of Psychosomatic Medicine and Psychotherapy, University of Leipzig Medical Center, Semmelweisstr. 10, 04103 Leipzig, Germany

**Keywords:** Childhood maltreatment, Maltreatment subtypes, Facial emotions, Attention, Emotion regulation strategy, Free-viewing task, Psychology, Risk factors

## Abstract

Little eye-tracking research has been conducted so far on the association of subtypes of childhood maltreatment and habitual emotion regulation with attention to emotions. To address this issue, gaze behavior of one hundred women with experiences of childhood maltreatment was examined in a free-viewing task in which pairs of faces consisting of an emotional (happy, surprised, angry, disgusted, fearful, and sad) and a neutral face were displayed. Participants’ childhood maltreatment experiences, emotion regulation strategies (reappraisal and suppression), anxiety, depression, stress perception, level of alexithymia, and intelligence were assessed. Fixation durations were used to assess attentional bias. A mixed linear model with post-hoc pairwise comparisons was used to investigate research questions. Results indicated that, at high levels of maltreatment severity, attentional preference for positive over other emotional faces was found to be diminished for physical and emotional abuse but not for emotional neglect. At a severe level of emotional abuse, a bias away from disgusted faces was observed. Suppression was linked to reduced and reappraisal was related to heightened attention allocation to facial emotions. Our data indicates that attention to social emotional information in adulthood could depend on the type and severity of childhood trauma and habitual emotion regulation strategy.

**Supplementary Information:**

The online version contains supplementary material available at 10.1038/s41598-025-99562-z.

## Introduction

Child maltreatment can comprise acts of omission like physical and emotional neglect and acts of commission like sexual, physical, and emotional abuse by a parent or a caregiver, which result in harm or potential harm to a child’s health, development, or dignity^1^. There is strong evidence that childhood maltreatment and early life trauma are associated with an increased risk of poor outcome in later life across a range of domains such as mental and physical health, social and academic functioning^2–4^. Experiences of childhood maltreatment are a leading contributor to the development of anxiety and depressive disorders among children and adults^5,6^. Childhood maltreatment is known to be linked to impairments in verbal and non-verbal intelligence in adulthood^7^. A growing body of literature addresses the effects of different types of abuse and neglect experiences during childhood on psychopathology and physical health^3,8,9^.

Repeated experiences of stress and violence in early developmental phases of neurocognitive development when children strongly depend on caregivers are assumed to lead to heightened sensitivity to threatening information and to have lasting effects on neural circuits underlying emotion processing and regulation^10,11^. Important factors, which may contribute to the development of emotional disorders and dysfunctions following childhood trauma, are biases in attention to threatening or other negative information. The dot-probe task is one of the most frequently used experimental tasks for assessing attention biases^12^. Using the dot-probe task, research on types of childhood maltreatment and negative attentional biases has yielded inconsistent results. In a sample of depressed patients, Günther et al.^13^ observed a positive association of emotional abuse and physical neglect with an attentional bias towards negative faces. In a sample of maltreated children, physical maltreatment (but not neglect) was linked to an attentional bias away from threatening faces^14^. Hori et al.^15^ found no correlations between subtypes of childhood maltreatment and attentional biases either toward or away from negative words. Finally, Blauth and Iffland^16^ reported associations of emotional abuse with increased attention toward angry faces and attentional avoidance of happy faces and associations of emotional neglect with attentional shifts toward happy faces and attentional avoidance of angry faces. The authors found no relation between physical abuse and attentional biases. In another study^17^, based on an evaluative conditioning task (combined with a face-in-the-crowd task) emotional abuse was linked to faster spatial detection of negatively associated faces in a crowd of faces. These mixed findings could be due to substantial differences in experimental methods, but also age, sex, and psychopathological status of study participants. Günther et al.^13^ examined inpatients (adult men and women) suffering from clinical depression, whereas Pine et al.^14^ compared a sample of maltreated children (boys and girls) suffering from anxiety symptoms and posttraumatic stress disorder with non-maltreated children; Blauth and Iffland^16^ examined a community sample of adults (men and women) suffering in part from mental disorders, and Hori et al.^15^ recruited a sample of healthy adult women. A serious methodological problem, which may also explain inconsistent findings, lies in the poor reliability (internal consistency and test–retest) of the attention parameters derived from the dot-probe task^18,19^. It has to be noted that none of the above-mentioned studies based on the dot-probe (or the evaluative conditioning/ face-in-the-crowd) task^13–17^ has provided information on reliability of the attentional bias scores. Reliability indicating the proportion of a measure’s variance, which reflects true score variance, is crucial for the ability to trust emerging findings. Reliable, psychometrically sound measures of attention are imperative for promoting our understanding of the attention processes, which might be implicated in psychopathology and childhood maltreatment^20,21^.

Eye-tracking technology is becoming increasingly important in psychological research to better understand attention processes during the perception of emotional contents^22,23^. Compared to reaction-time based tasks, eye-tracking systems allow a direct and continuous measurement of visual attention^24^. For example, it is possible to examine which face in a pair of faces is looked at longer or how fast a face is fixated. Indices of attentional bias based on fixation duration manifest adequate to good reliability for free viewing tasks^25,26^. In a recent eye-tracking study^27^, early and late processes of attention allocation to happy, sad, and disgusted facial expressions were examined as a function of experienced childhood maltreatment (based on sum scores) in a sample of healthy women. Childhood maltreatment was related to faster gaze shifts towards emotional faces and shorter dwell time on disgusted faces. These results indicate a vigilance-avoidance pattern of attention allocation associated with childhood maltreatment. It was hypothesized that poor abilities in regulating negative emotions may underlie the observed avoidance at a late stage of processing^27^. It remains to be clarified which types of maltreatment are associated with late avoidance of hostile faces.

Experiences of maltreatment in childhood are connected with increased habitual expressive suppression and reduced habitual reappraisal in adults^28^. Cognitive reappraisal and expressive suppression are two of the most studied emotion regulation strategies^29^. These strategies can be understood in a framework, which conceptualizes emotional processing and reacting as a sequence, beginning with perceptual processes, followed by interpretation processes, and then a drive to respond with action^30^. Within this approach, reappraisal involves an attempt to reinterpret stimuli or events in a more positive way, whereas suppression refers to the inhibition of behavioral expressive responses to emotional stimuli^31^. Cognitive reappraisal is assumed to operate earlier than suppression in the sequence of emotional processing events^32^. Both strategies refer to the regulation of positive and negative emotions. Numerous studies have shown that in general cognitive reappraisal is associated with adaptive outcomes while expressive suppression is related to maladaptive outcomes in mental health, and social functioning^33,34^. Gross and John^31^ developed the *Emotion Regulation Questionnaire* (ERQ) to assess the habitual use of cognitive reappraisal and expressive suppression.

Hitherto, there has been little research on the impact of habitual use of cognitive reappraisal and expressive suppression on attention allocation to emotional information. In an eye-tracking study based on a free-viewing task, Bardeen and Daniel^35^ explored the links between use of cognitive reappraisal and expressive suppression as regulatory strategies in everyday life and attentional bias to threat using threatening and neutral pictorial stimuli from the International Affective Picture System (IAPS^36^). Expressive suppression predicted heightened attentional bias to threat images in pairs of neutral and threat-related images (using dwell times), but only at lower levels of cognitive reappraisal. It was concluded that those who habitually use expressive suppression to the exclusion of other regulatory strategies could be at risk to exhibit increased attention to threat information. In a subsequent eye-tracking study, Dolcos et al.^37^ investigated the effectiveness of self-guided focused attention in reducing the impact of negative pictures on experienced negative affect. A series of composite images with distinguishable foreground (either negative or neutral) and background areas (always neutral) was administered with the task to focus on the foreground or background content. It was observed that increased effectiveness in focusing on neutral contents was linked to habitual use of suppression as an emotion regulation strategy^37^. The connection between attentional avoidance of negative information and suppression seems reasonable given that both involve inhibitory processes, and their execution appears linked to similar neural responses in top-down cortical regions such as dorsolateral prefrontal areas^38,39^. According to Dolcos et al.^37^ suppression could be a type of “avoidance strategy”, which may provide immediate benefits concerning the downregulation of negative affects (see also^40^).

In the present eye-tracking study, we had two main research questions. First, we examined the association between subtypes of childhood maltreatment and allocation of attention to various facial emotions at a late processing stage. The present investigation could help to specify which type and at what level childhood maltreatment experiences are related to attentional biases toward or away from social emotional stimuli. In our study, we recruited individuals with experiences of childhood maltreatment, which could consist of experiences of abuse (physical, emotional, and/or sexual) and/or of neglect (physical and/or emotional). Through this recruitment approach, we could examine whether dosage of specific maltreatment experiences is connected with attention allocation to facial emotions. To investigate the effect of dosage we used the classification of maltreatment severity as proposed by Bernstein and Fink^41^, which consists of four levels of severity for each trauma type: none (to minimal), low (to moderate), moderate (to severe), and severe (to extreme). We also explored the effect of overall childhood maltreatment severity on attention allocation to faces of different emotional qualities.

Second, we examined whether the emotion regulation strategies of expressive suppression and cognitive reappraisal are related to late processes of attention allocation to emotional facial expressions in individuals with a history of childhood maltreatment. Since sex differences in attentional biases toward emotional faces^42^ and in recognizing facially expressed emotions have been reported^43,44^ we included only women in the present study and examined the effects of childhood maltreatment and emotion regulation strategy in a homogeneous sample concerning biological sex.

To assess attention allocation, we administered a free-viewing task in which pairs of facial stimuli were shown simultaneously: an emotional (i.e., happy, surprised, angry, disgusted, fearful, or sad) and a neutral expression of the same model. We based our analysis of eye-movements on the time participants’ gaze fixated on the emotional in comparison with the neutral face as an index of late attentional preference. Because in our previous study^27^, which was based on an independent sample, we had observed a negative relationship between childhood maltreatment (based on sum scores), and dwell time on disgusted faces at a late processing stage we expected that abusive forms of maltreatment would be associated with decreased sustained attention to hostile or threat-related facial expressions, i.e., angry, and disgusted faces. Experiences of emotional neglect during childhood mean that caregivers did not pay attention to the child’s emotional states and it seems likely that there was little emotional exchange with caregivers. This lack of learning experiences could have led to an impoverished habitual attention to one’s own emotions and those of others. There is evidence that emotional neglect during childhood is linked to alexithymic personality traits in adulthood^45,46^. Against this background, we hypothesized that emotional neglect could be related to less attention to emotional information at a late processing stage. Finally, since the findings of previous studies on the emotion regulation strategy of expressive suppression and attention to emotional stimuli are inconsistent^35,37^, we stated non-directional hypotheses and assumed that expressive suppression, unlike cognitive reappraisal, would be related to attention allocation to emotional faces.

## Results

### Relations of childhood maltreatment with emotion regulation, anxiety, depression, stress perception, alexithymia, and intelligence

Table 1 shows the correlations between the CTQ total and subscale scores and the other psychometric variables.

**Table 1:** Descriptive statistics of and correlations between self-report scales and tests (N = 100) with Cronbach’s alpha for self-report questionnaires.

Variable	1	2	3	4	5	6	7	8	9	10	11	α
1. CTQ												0.82
2. CTQ-PA	0.61***											0.79
3. CTQ-EA	0.71***	0.39***										0.73
4. CTQ-SA	0.42***	0.16	− 0.02									0.86
5. CTQ-EN	0.68***	0.11	0.41***	0.07								0.84
6. ERQ-Re	− 0.01	− 0.07	− 0.08	0.26**	− 0.07							0.71
7. ERQ-Su	0.27**	0.06	0.30**	− 0.15	0.28**	− 0.04						0.75
8. STAI T	0.20*	0.12	0.26**	− 0.20*	0.23*	− 0.34***	0.39***					0.90
9. BDI-II	0.25*	0.21*	0.20*	− 0.06	0.24*	− 0.18	0.28**	0.78***				0.87
10. PSS-10	0.07	0.11	0.16	− 0.33***	0.14	− 0.24*	0.27**	0.70***	0.67***			0.83
11. TAS-20	0.19	0.06	0.20*	− 0.18	0.27**	− 0.13	0.45***	0.45***	0.44***	0.45***		0.85
12. MWT	− 0.09	− 0.18	− 0.11	0.09	0.00	− 0.18	− 0.18	− 0.15	− 0.06	− 0.18	− 0.19	–

	1	2	3	4	5	6	7	8	9	10	11	12
Mean	58.30	7.84	15.62	6.83	18.18	25.95	14.80	49.91	16.24	31.88	47.46	106.24
SD	11.60	3.95	4.40	3.82	3.91	6.61	5.09	10.03	8.40	5.73	11.82	9.42

*CTQ* Childhood trauma questionnaire, *CTQ-PA* scale physical abuse, *CTQ-EA* scale emotional abuse, *CTQ-SA* scale sexual abuse, *CTQ-EN* scale emotional neglect, *ERQ-Re* scale cognitive reappraisal of the Emotion regulation questionnaire, *ERQ-Su* scale expressive suppression of the Emotion regulation questionnaire, *STAI T* State trait anxiety inventory, trait version, *BDI-II* Beck depression inventory, *PSS-10* Perceived stress scale, *TAS-20* 20-item Toronto alexithymia scale, *MWT* Mehrfachwahl-Wortschatz-Intelligenztest version B, intelligence quotient.

*p < 0.05 (two-tailed), **p < 0.01 (two-tailed), ***p < 0.001 (two-tailed).

The analysis yielded significant positive correlations between the CTQ total score and expressive suppression, trait anxiety, and depressive symptoms. The subscales emotional abuse and emotional neglect correlated positively with expressive suppression, trait anxiety, level of depressive symptoms, and alexithymia. Physical abuse showed only a correlation with depressive symptoms. Sexual abuse was positively related to cognitive reappraisal and negatively related to trait anxiety and perceived stress during the past month. Moreover, cognitive reappraisal was negatively related to trait anxiety and perceived stress, whereas expressive suppression was positively associated with trait anxiety, depressive symptoms, perceived stress, and alexithymia (see Table 1 for details). Intelligence was not correlated with any of the questionnaire measures.

### Free-viewing task

#### Reliability of the free-viewing task

Our reliability analysis yielded high reliability coefficients for the total fixation duration bias score. Using 500 random splits, the Spearman-Brown corrected split-half reliability estimates were above 0.9 for all emotion conditions (see Supplementary Table S1 for Spearman–Brown and Split-half reliability coefficients).

#### Fixation duration bias

Mean fixation durations for emotional and neutral expressions are shown in Supplementary Fig. S1. Paired sample t-tests were performed to assess the difference between total fixation duration on the emotional and paired neutral facial expressions. Across all emotion conditions, total fixation duration on emotional facial expressions was significantly higher than on paired neutral facial expressions (see Supplementary Table S2 for t-test results).

#### The relationship of fixation duration bias with type and level of childhood maltreatment and emotion regulation styles

**Fig. 1:**
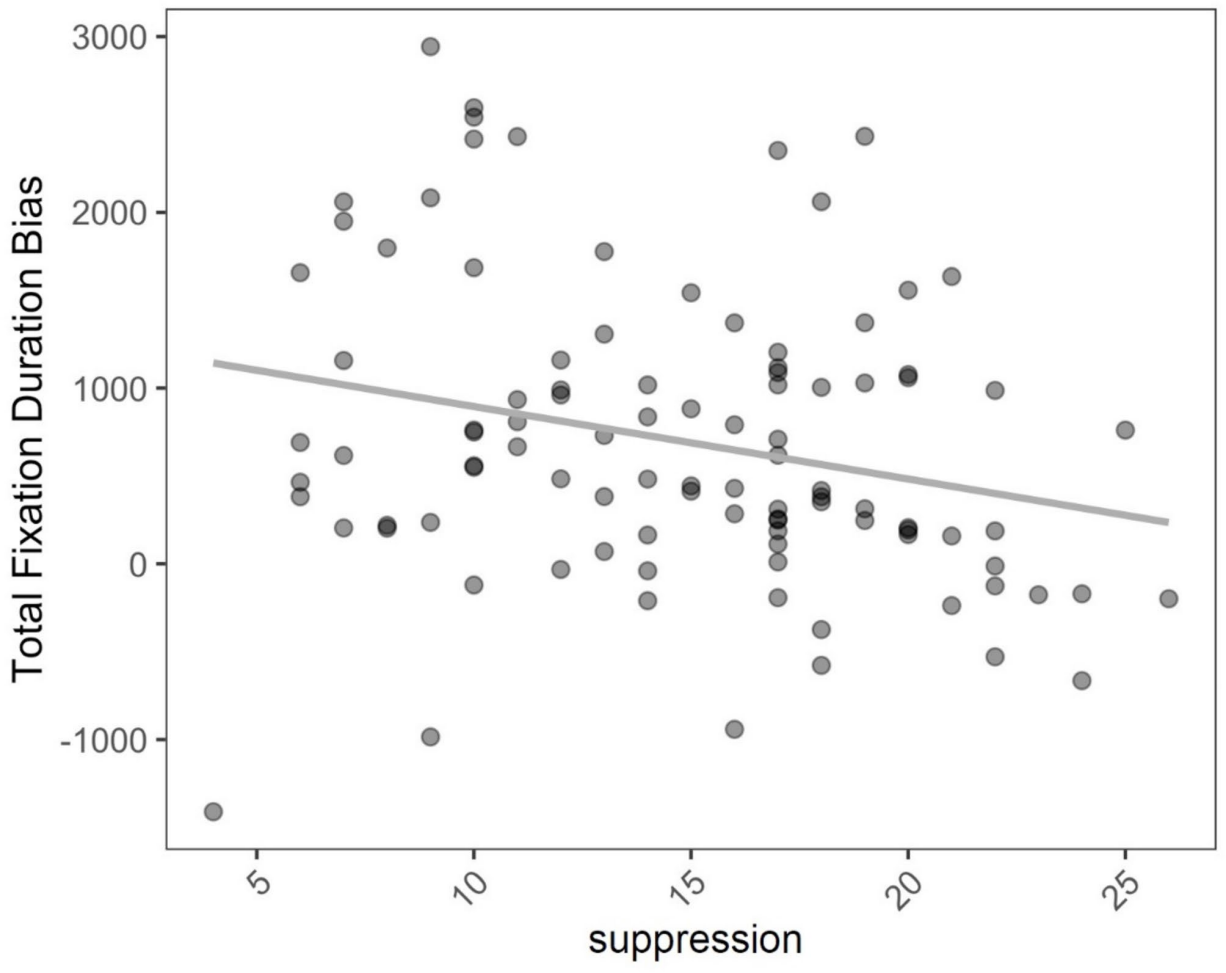
Negative association between expressive suppression and overall fixation duration bias for emotional faces.

The results of our mixed linear model showed no significant main effects for type of childhood maltreatment (i.e., physical, sexual, and emotional abuse and emotional neglect). Moreover, no main effects of the level of depressive symptoms, trait anxiety, alexithymia, and current stress experience were observed. However, our results indicated a significant main effect of expressive suppression, *F* (1,84) = 8.83, *p* = 0.004, with higher values going along with decreased attentional bias scores (see Fig. 1). Cognitive reappraisal also had a significant effect on attentional bias scores, *F* (1,84) = 6.02, *p* = 0.016, with greater scores on the cognitive reappraisal scale being associated with increased attentional bias to emotional faces. We also found an effect of position, *F* (1,11,869) = 4.36, *p* = 0.03, indicating that emotional faces that were displayed on the left side were looked at longer. Finally, the results of our mixed linear model indicated a significant main effect of emotion, *F* (5,11,869) = 8.74, *p* < 0.0001, and three significant interactions between emotion and type of childhood maltreatment, for physical abuse, *F* (5,11,869) = 3.51, *p* = 0.004, emotional abuse, *F* (5,11,869) = 2.81, *p* = 0.015, and emotional neglect *F* (5,11,869) = 2.38, *p* = 0.036.

#### Physical abuse: differences in fixation duration bias between emotions as a function of severity level

**Fig. 2:**
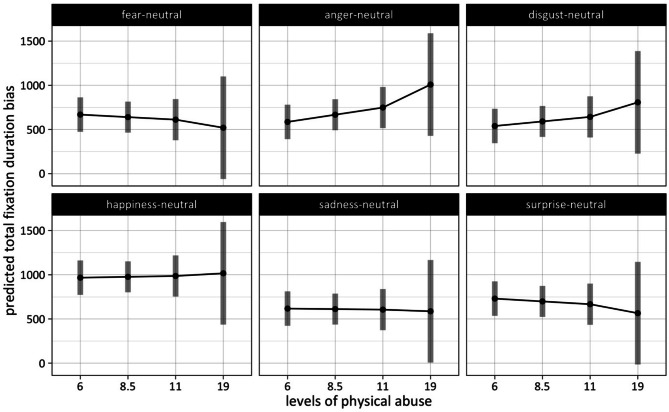
Fixation duration bias on emotional faces as a function of level of physical abuse (numbers on the x-axis represent the median of scores within a severity level of physical abuse). The number of participants per severity level was 65 (for none or “6”), 12 (for low or “8.5”), 10 (for moderate or “11”), and 13 (for severe or “19”).

We performed pairwise comparisons among the marginal means of the emotion conditions at each level of physical abuse (PA) (estimated marginal means are presented in Supplementary Table S3). At the lowest level, total fixation duration bias is the highest in the happiness condition (*M* = 967 ms, *SE* = 98 ms) and the lowest in the disgust condition (*M* = 540 ms, *SE* = 98 ms). We found significant differences between happiness and every other emotional condition, respectively. On the other end, disgust with the lowest attentional bias at this level of physical abuse also reaches a statistically significant difference compared to the surprise condition (*M* = 730 ms, *SE* = 98 ms) (see Fig. 2).

The statistically significant difference between the happiness and the other emotion conditions persists for the middle levels (2 and 3) of physical abuse (PA level 2: *M* = 976 ms*, SE* = 88 ms; *PA level 3: M* = 986 ms, *SE* = 117 ms), with an increased estimated total fixation duration bias in the happiness condition compared to all other emotion conditions. For the highest level of physical abuse (level 4) happiness (*M* = 1016 ms, *SE* = 293 ms) shares the highest total fixation duration on emotional faces with anger (*M* = 1008 ms, *SE* = 293 ms). At the bottom end, total fixation duration bias in the fear condition shows the lowest attentional bias (*M* = 520 ms, *SE* = 293 ms) of all emotions. This leaves only the difference in total fixation duration bias between fear and anger, and fear and happiness, respectively significant (see Fig. 2) (see for statistical details on these contrasts Supplementary Table S4).

#### Emotional abuse: differences in fixation duration bias between emotions as a function of severity level

For the lowest level of emotional abuse (EA), happiness (*M* = 1143 ms, *SE* = 238 ms) displayed the highest total fixation duration bias of all emotions, while sadness (*M* = 462 ms, *SE* = 238 ms) was the condition with the lowest attentional bias (estimated marginal means are presented in Supplementary Table S5). Pairwise comparisons revealed that all conditions but the disgust condition (*M* = 832 ms, *SE* = 238 ms) differed significantly from the happiness condition at the lowest level of emotional abuse (see for statistical details on these contrasts Supplementary Table S6).

For both middle levels this pattern persists, with the happiness condition displaying the highest total fixation duration bias (*EA level 2: M* = 1069 ms, *SE* = 152 ms; *EA level 3: *1004 ms, *SE* = 95 ms) and sadness being at the lower end (*EA* = *level 2: M* = 528 ms, *SE* = 152; *EA level 3: *586 ms, *SE* = 95 ms)*.* Again, bias scores in all emotion conditions differed significantly from the happiness condition. No other pairwise comparisons reached statistical significance.

**Fig. 3:**
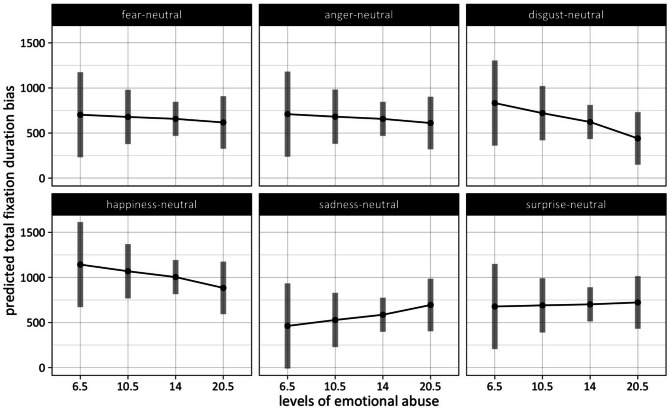
Fixation duration bias on emotional faces as a function of level of emotional abuse (numbers on the x-axis represent the median of scores within a severity level of emotional abuse). The number of participants per severity level was 5 (for none or “6.5”), 22 (for low or “10.5”), 21 (for moderate or “14”), and 52 (for severe or “20.5”).

Lastly, for the highest level of emotional abuse, happiness (*M* = 884 ms, *SE* = 147 ms) continues to be at the top. But, instead of sadness, it’s the disgust condition (*M* = 441 ms, *SE* = 147 ms) that displays the lowest total fixation duration bias of all emotions here. At the highest level of emotional abuse, we found significant differences between the happiness condition and anger (*M* = 611 ms, *SE* = 147 ms), fear (*M* = 617 ms, *SE* = 147 ms), and disgust, respectively. Furthermore, we found significant differences between disgust and sadness (*M* = 694 ms, *SE* = 147 ms) and disgust and surprise (*M* = 723 ms, *SE* = 147 ms (see Fig. 3))*.*

#### Emotional neglect: differences in fixation duration bias between emotions as a function of severity level

**Fig. 4:**
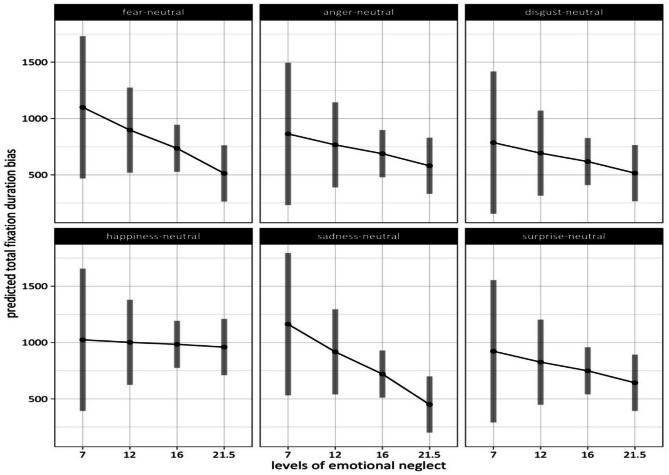
Fixation duration bias on emotional faces as a function of level of emotional neglect (numbers on the x-axis represent the median of scores within a severity level of emotional neglect). The number of participants per severity level was 3 (for none or “7”), 13 (for low or “12”), 20 (for moderate or “16”), and 64 (for severe or “21.5”).

We conducted pairwise comparisons to assess the interaction effect between the CTQ subscale emotional neglect (EN) and the emotion conditions. At the lowest level of emotional neglect, sadness (*M* = 1163 ms, *SE* = 318 ms) shows the highest total fixation duration bias amongst the emotion conditions, while disgust (*M* = 786 ms, *SE* = 318 ms) shows the lowest (estimated marginal means are presented in Supplementary Table S7). Yet, all estimated marginal means show a high variance (see Fig. 4), resulting in no statistically significant differences among them (see for statistical details on these contrasts Supplementary Table S8).

For the next higher level of emotional neglect, total fixation duration bias was highest for happiness (*M* = 1002 ms*, SE* = 190 ms) and lowest for disgust (*M* = 692 ms*, SE* = 190 ms). Only the difference between these two conditions reaches statistical significance at this level.

The estimated marginal means at level 3 of emotional neglect, also revealed happiness (*M* = 984 ms,* SE* = 105 ms) as condition with the highest attentional bias score and disgust (*M* = *618* ms, *SE* = 105 ms) as condition with the lowest. Here, pairwise comparisons between happiness and all other emotion conditions, respectively, were significant, but no other comparisons between emotions conditions were significant.

At the highest level of emotional neglect, happiness (*M* = *959* ms, *SE* = 126 ms) persists to have the highest attentional bias score, but sadness (*M* = 450 ms, *SE* = 126 ms) has replaced disgust (*M* = 515 ms, *SE* = 126 ms) as condition with the lowest attentional bias score. Again, all differences between happiness and the remaining emotion conditions reached statistical significance, while no other pairs were significantly different from each other (see Fig. 4).

#### The relationship of fixation duration bias with overall childhood maltreatment severity

The results of our second mixed linear model indicated no significant main effects for overall childhood maltreatment (as assessed by the CTQ total score), level of depressive symptoms, trait anxiety, alexithymia, and current stress experience. Not surprisingly, as in the first linear model (see above) the analysis showed a significant main effect of expressive suppression, *F* (1,92) = 8.25, *p* = 0.005, with higher values going along with decreased attentional bias scores. In addition, there was a main effect of cognitive reappraisal on attentional bias scores, *F* (1,92) = 5.59, *p* = 0.02, with greater reappraisal scores being associated with increased attentional bias to emotion faces. There was also an effect of position, *F* (1,11,889) = 4.34, *p* = 0.04, indicating that emotional faces presented on the left side were looked at longer. Lastly, the results of the second linear model showed a main effect of emotion, *F* (5,11,889) = 10.88, *p* < 0.0001, and a significant interaction between emotion and overall childhood maltreatment, *F* (5,11,889) = 7.63, *p* < 0.001.

#### Overall childhood maltreatment severity: differences in fixation duration bias between emotions as a function of severity level

**Fig. 5:**
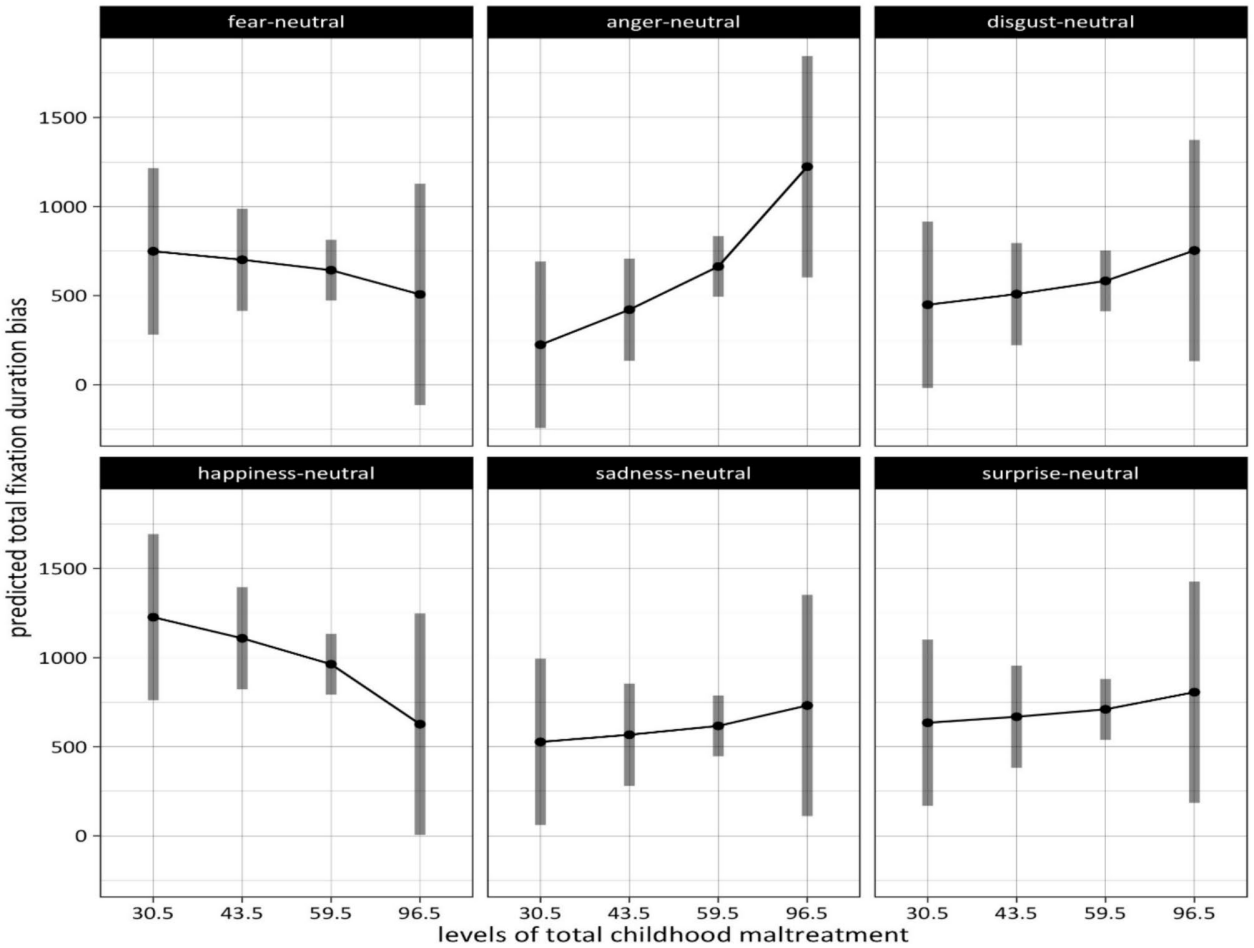
Fixation duration bias on emotional faces as a function of level of overall childhood maltreatment (numbers on the x-axis represent the median of scores within a severity level of overall maltreatment). The number of participants per severity level was 2 (for none or “30.5”), 28 (for low or “43.5”), 52 (for moderate or “59.5”), and 18 (for severe or “96.5”).

To assess the interaction effect between overall childhood maltreatment and the emotion conditions we performed pairwise comparisons (estimated marginal means are presented in Supplementary Table S9). At the lowest level, total fixation duration bias is the highest in the happiness condition (*M* = 1227 ms, *SE* = 238 ms) and the lowest in the anger condition (*M* = 224 ms, *SE* = 238 ms). We found significant differences between happiness and every other emotion condition (see for statistical details on the contrasts Supplementary Table S10). On the other end, anger with the lowest attentional bias at this level of overall maltreatment also reaches statistically significant differences compared to the fear (*M* = *749 ms, SE* = *238 ms*) and the surprise condition (*M* = 634 ms*, SE* = 238 ms) (see Fig. 5).

The significant difference between the happiness and all other emotion conditions persists for both middle severity levels (2 and 3) of overall maltreatment (level 2: *M* = 1109 ms, *SE* = 146 ms; level *3: M* = 963 ms, *SE* = *87* ms), with an increased estimated total fixation duration bias in the happiness condition compared to all other emotion conditions. At severity level 2, total fixation duration bias is lowest in the anger condition (*M* = *421* ms, *SE* = 146 ms) and shows significant differences compared to that in the fear (*M* = 702 ms*, SE* = 146 ms) and surprise condition (*M* = 668 ms*, SE* = 146 ms) (see Fig. 5).

For the highest level of overall maltreatment (level 4) total fixation duration bias is the highest in the anger condition (*M* = 1224 ms*, SE* = 317 ms) and lowest in the fear (*M* = 507 ms, *SE* = 317 ms), happiness (*M* = 627 ms, *SE* = 317 ms), and sadness condition (*M* = 731 ms, *SE* = 317 ms). At the highest level of overall maltreatment, total fixation duration bias is higher for anger than for fear, happiness, and sadness. No other significant differences between emotion conditions were observed (see for statistical details on these contrasts Supplementary Table S10).

## Discussion

A central aim of our eye-tracking study was to examine the association between subtypes of childhood maltreatment and allocation of attention to different qualities of facial emotions at a late processing stage. We recruited women with experiences of childhood maltreatment that consisted of experiences of abuse and/or of neglect. According to the classification of maltreatment severity proposed by Bernstein and Fink^41^, our study participants reported to have frequently experienced emotional neglect (64%) and emotional abuse (52%) during childhood at a severe level. Many study participants indicated to have not suffered from any physical or sexual abuse (65% and 67% respectively). Since the subscale physical neglect manifested insufficient internal consistency in our sample, we did not report its results here. To sum up, in our sample severe maltreatment experiences were rather frequent for emotional neglect and emotional abuse but severe maltreatment experiences were relatively rare for physical and sexual abuse.

We hypothesized that abusive forms of maltreatment are related to decreased sustained attention to threat-related, hostile facial expressions (i.e., angry, and disgusted faces). According to the results of a repeated measure linear mixed model no main effects were found for types of childhood maltreatment (i.e., physical, sexual, and emotional abuse and emotional neglect). Our findings revealed interactions of emotion and type of childhood maltreatment for physical abuse, emotional abuse, and emotional neglect. Post hoc analyses based on pairwise comparisons of estimated marginal means showed evidence for a positive attentional bias, i.e., happy faces were fixated longer than the other emotion faces (compared to neutral faces), for the first three severity levels of physical abuse (i.e., none to moderate maltreatment experiences). At the most severe level of physical abuse this attentional prioritization of facial happiness was largely lost. Instead, facial anger received more attention at this level (significantly more than facial fear). Thus, severe experiences of physical abuse appear to be related to a substantial reduction in attentional prioritization of positive facial expressions and an attentional tendency towards angry facial expressions, which is not observed at lower severity levels of physical abuse. Thus, our above-mentioned hypothesis was not supported for physical abuse. We found no evidence that physical abuse is related to decreased sustained attention to angry or disgusted faces.

The results of further post hoc analyses indicate a somewhat different picture of attention allocation for emotional abuse. At a low and moderate severity level of emotional abuse study participants exhibited a clear positive attentional bias, fixating happy faces significantly longer than the other emotion faces. At the highest severity level, the positive attention prioritization of positive faces was less pronounced, as happy faces were fixated longer than fearful, angry, and disgusted faces (but as long as sad and surprised faces—compared to neutral faces). At a severe level of emotional abuse facial disgust showed the lowest fixation duration bias score, which was significantly lower than those for sadness and surprise. This means that severe experiences of emotional abuse seem to be related to a partial reduction in attentional prioritization of positive facial expressions and attentional tendencies away from disgusted facial expressions, which is not found at lower severity levels of emotional abuse. Thus, in our study we observed at least some support for the hypothesis that emotional abuse is related to decreased sustained attention to disgusted faces. However, there was no evidence that emotional abuse is related to decreased sustained attention to angry facial expressions.

According to the results of post hoc analyses for emotional neglect, we found a clear positive attentional bias, fixating happy faces significantly longer than the other emotion faces, at a moderate and severe maltreatment level. At a modest severity level of emotional neglect, the positive attention prioritization of positive faces was less pronounced, as happy faces were only fixated longer than disgusted faces. At the lowest severity level, no differences in fixation duration bias scores were revealed between emotion categories. As the number of participants with no or moderate emotional neglect experiences was low in our study (n = 3 and n = 13, respectively), one should be cautious in drawing conclusions about the (relative) absence of a positive attentional bias at low severity levels of emotional neglect. However, it is interesting to note that high severity levels of emotional neglect seem to be associated with a clear attentional prioritization of positive over negative faces. Importantly, our hypothesis that emotional neglect is related to less attention to emotional information at a late processing stage was not supported by our data. There was no evidence that little emotional exchange with caregivers during childhood goes along with reduced attention allocation to other persons’ facial emotions.

To summarize, our data indicates that sustained attention allocation varies by type and severity of childhood trauma experienced. At high levels of maltreatment severity attentional preference for positive compared to other emotional facial expressions is diminished for physical abuse and to a lesser extent for emotional abuse. In contrast, at high levels of emotional neglect no evidence was found for a decreased positive attentional bias. Loss or reductions of positive attentional biases occur in clinical depression^47,48^ and dysphoria^49^. Healthy individuals direct their attention to positive information to repair negative moods^50^. It has been shown that all types of childhood maltreatment increase the risk of developing depressive disorders^5,51^. Reductions in attentional preference for positive stimuli may be one pathway through which experiences of severe physical and emotional abuse heighten the risk for the development of negative affect and depressive symptoms.

Our result of a bias away from disgusted facial expressions at a severe level of emotional abuse confirms and specifies the finding of Hoepfel et al.^27^ who reported a negative relationship between overall childhood maltreatment load (CTQ sum score), and dwell time on disgusted faces at a late processing stage. Severely emotionally abused individuals could experience disgusted faces as especially aversive and tend to avoid them. Disgust is a hostility-related emotion indicating a request to increase interpersonal distance in human interactions^52^. The facial expression of disgust signals revulsion and social disapproval^53^ and could play a central role in maltreating children emotionally, by humiliating and devaluating them.

In our study, we also investigated the effect of overall childhood maltreatment on attention allocation to facial emotions. According to our data, there was a positive attentional bias, i.e., happy faces were looked at longer than the other emotion faces (compared to neutral faces), for the first three severity levels of overall childhood maltreatment experience, i.e., none to moderate maltreatment severity. At the most severe level of overall childhood maltreatment this attentional prioritization of facial happiness was completely lost. Instead, facial anger received most attention at this level (significantly more than facial fear, happiness, and surprise). This means that at high levels of traumatic load, which also implies experiences of multiple forms of maltreatment, the pattern of sustained attentional orientation towards socio-emotional signals might be fundamentally changed compared to low and middle severity levels of childhood maltreatment. The attentional preference for positive facial expressions could be lost and, at least in part, replaced by an attentional preference for angry facial expressions. It is interesting to note that, in contrast, at minimal and moderate levels of childhood maltreatment, angry faces tend to be avoided. An increased attention to social threat signals and a diminished attention to positive facial expressions may substantially heighten the risk of developing anxiety and depressive disorders in people with severe experiences of childhood maltreatment.

A second aim of the present study was to investigate whether the emotion regulation strategies of expressive suppression and cognitive reappraisal are linked to late processes of attention allocation to facial emotions in individuals with a history of childhood maltreatment. So far, there has been little research on the effect of habitual use of suppression and reappraisal on attention allocation to emotional information. Both strategies refer to the regulation of negative and positive emotions^30,31^. It is known that experiences of maltreatment in childhood are connected to increased habitual expressive suppression and reduced habitual reappraisal in adults^28^. According to our results expressive suppression as well as cognitive reappraisal had an impact on attention allocation to facial emotions. Expressive suppression was negatively associated whereas cognitive reappraisal was positively linked to attention to emotional faces. This means that habitual inhibition of behavioral expressive responses to emotional stimuli seems to go along with more attention to neutral and less attention to emotional faces. The present data supports our (non-directional) hypothesis that suppression is related to attention allocation to emotional faces. Our observation is in line with the finding of Dolcos et al.^37^ who observed that habitual use of suppression as an emotion regulation strategy is related to an increased effectiveness in focusing on neutral information when looking at composite images with negative and neutral contents. Suppression could represent a type of general “avoidance strategy” concerning emotions at a perceptual and expressive level, which could have benefits by making the development of negative affect through emotional contagion less likely. The connection between expressive suppression and attentional avoidance of emotional information might be explained by the fact that both involve processes of inhibition, and their execution could be based, at least in part, on similar neural structures in the prefrontal cortex^38,39^.

Contrary to our expectations, we found cognitive reappraisal to be associated with processes of attention to emotional faces. It appears that individuals high in habitual reappraisal pay more attention to social emotional information, regardless of valence. Such a pattern of attention allocation could favor processing and reinterpretation of emotional stimuli. The present results highlight the importance of considering attention mechanisms in understanding habitual cognitive reappraisal. The extent to which emotions are attended to has a positive impact on emotion clarity and promotes building up of emotion knowledge^54,55^. High emotional clarity in turn appears to be related to a more frequent use of reappraisal^56,57^.

In our study, we administered a free-viewing paradigm with emotional-neutral face pairs to assess late processes of attention allocation. The application of a free-viewing task allowed us to investigate self-generated, endogenously controlled gaze behavior^58^. Our analysis of gaze behavior was based on the time participants’ gaze fixated on the emotional in comparison with the neutral face. Across all emotion conditions, high reliability coefficients were observed for the fixation duration bias scores. This fits in well with previous eye-tracking research based on the free-viewing task showing adequate to good reliability for indices of attentional bias based on dwell time or fixation duration^59^. It is known that the free-viewing paradigm does not provide a reliable means to study early attentional biases^26,60^.

Though the present study had strengths, several limitations should be acknowledged. In our free-viewing task we administered pairs of faces in which an emotional expression was always combined with a neutral one. It can be criticized that neutral facial expressions were used as baseline condition. Prototypical neutral faces are characterized by no facial muscle contraction^61^ and, at first glance, one may assume that these faces appear unemotional, not conveying an affective message^62^. However, individuals with high compared to low levels of childhood maltreatment appear to be particularly prone to misinterpret neutral facial expressions as negative^63,64^. Against this background, it might be advisable to administer mildly happy faces instead of or in addition to neutral faces as baseline condition (e.g., 10–25% morphs along the neutral to happy continuum) in future studies on childhood maltreatment and attention to emotions using tasks with pairs of faces. We recruited primarily young, well-educated white/caucasian women with various types of childhood maltreatment experiences (but mainly emotional neglect and emotional abuse) as study participants, which clearly limits the generalizability of our findings. Future research is necessary to clarify the relation of sexual abuse and physical neglect with attention to social emotional information. It is an important task to examine the associations of exposure to childhood trauma and attention to facial emotions in men since sex differences were observed in maltreated individuals concerning affective reactions to emotional images suggesting that adverse childhood experiences (especially emotional neglect and physical maltreatment) could make men more emotionally responsive in their adulthood than women^65^. Furthermore, it has to be clarified whether there exist relations of expressive suppression and cognitive reappraisal with attention to facial emotions in healthy women (and men) without trauma experiences during childhood. It can be criticized that assessment of childhood maltreatment was exclusively based on a retrospective, self-report measure, which could have led to distortions in recall. Moreover, despite its common use the CTQ has been criticized for neglecting important timing information of maltreatment^66^. Future studies may administer the *Maltreatment and Abuse Chronology of Exposure* (MACE) Scale, which allows a retrospective assessment of ten types of maltreatment during each year of childhood^66,67^.

## Materials and methods

### Participants

Our final sample included one hundred women, from age 18 to 30, with a mean age of 23.15 years (*SD* = 3.33). Participants were recruited through both social media and traditional methods (e.g., by posting recruitment ads on noticeboards) at the Universities of Leipzig and Halle. Most participants were students (91%). All participants were German native speakers or spoke German since the age of six. They were screened for multiple exclusion criteria before partaking in the study. Exclusion criteria were any self-reported previous or current mental or neurological illnesses, especially current presence of depressive episodes, abuse of alcohol or other substances, pregnancy, and compromised vision. To exclude the current presence of a major depressive episode we explicitly asked participants whether they felt sad, empty, or hopeless most of the day for a 2-week period in the past month or whether they lost interest or enjoyment in activities over a 2-week period in the last month. All participants reported to have experienced at least one type of childhood maltreatment (physical abuse, emotional abuse, sexual abuse, physical neglect, and/or emotional neglect). The self-reported information was objectified by the CTQ data: according to the severity classification of Bernstein and Fink^41^ all study participants had at least one type of maltreatment experience at level 2 “low to moderate” during childhood. Considering the level 2 (“low to moderate”) criterion of severity^41^, two of our participants experienced one type of maltreatment, 16 participants experienced two maltreatment types, 38 participants experienced three maltreatment types, 29 participants experienced four maltreatment types, and 15 participants experienced five maltreatment types. All subjects gave written informed consent before study participation. The study was conducted in accordance with the Declaration of Helsinki^68^ and approved by the ethics committee of the Medical School at the University of Leipzig.

### Self-report questionnaires

We measured participants’ mood and dispositional anxiety because the level of depressive symptoms and trait anxiety have been found to be linked to heightened attention to negative stimuli^69,70^. As the personality trait alexithymia is known to affect processes of emotion face perception^71,72^ we decided to also assess and control alexithymia in our investigation. Participants’ intelligence was measured in our study because general intelligence can be related to a more efficient attentional processing of emotional information^73^. Finally, participants’ level of perceived stress during the past month was measured since psychological stress seems to promote attentional control in selective attention processes^74^.

#### Childhood maltreatment

The German version of the *Childhood Trauma Questionnaire* (CTQ^75^) was used to assess traumatic childhood experiences. The CTQ consists of five subscales: emotional abuse, physical abuse, sexual abuse, emotional neglect, and physical neglect. Each subscale comprises 5 items, which are rated on a 5-point scale. Subscale scores of the CTQ can range from 5 to 25. In the present study, the CTQ subscale physical neglect was not included in the statistical analyses because it showed an insufficient internal consistency (Cronbach’s alpha: 0.54) in our sample. A prior study on the validity of the German adaptation of the CTQ^76^ observed also a low internal consistency of the subscale physical neglect and cautioned against the use of the scale. In our investigation, the subscale physical neglect was used in forming the total score of the CTQ. Internal consistency and retest-reliability are acceptable to high for the CTQ scales (except physical neglect)^75^. There is evidence for factorial and concurrent validity of the CTQ^77,78^. Longitudinal data over a 2-year period suggest that the CTQ provides temporally stable self-reports of childhood maltreatment in healthy and clinical populations^79^.

#### Anxiety

The trait version of the *State-Trait Anxiety Inventory* (STAI^80^) measured participants’ dispositional anxiety. The STAI consists of 20 items, which are evaluated on a 4-point scale. The sum score varies between 20 and 80. Internal consistency of the STAI trait version was found to be high (α > 0.80), and retest-reliability over a 2-month period exceeds r > 0.77 in nonclinical student samples^80^. Moreover, there is evidence of concurrent and divergent validity for the trait version of the STAI^80,81^.

#### Depression

The *Beck Depression Inventory* (BDI-II^82^) assesses presence and severity of depressive symptoms during the preceding 2 weeks. The BDI-II comprises 21 items relating to symptoms of depression such as negative cognitions, hopelessness, and physical symptoms. Every item of the BDI-II consists of four response options, which are graded by severity. The sum score can vary between 0 and 63. Internal consistency of the BDI-II was found to be satisfactorily high (α > 0.84), and retest-reliability exceeds r > 0.75 in nonclinical samples^83^. Associations with construct-related scales (depression, dysfunctional cognitive constructs) were found to be high^83^.

#### Stress

The *Perceived Stress Scale* (PSS-10^84^) was administered to assess the extent to which participants experience their lives as stressful during the last month. The PSS-10 consists of 10 items, which are rated on a 5-point Likert scale. The sum score ranges between 10 and 50. Internal consistency of the total PSS-10 score is good in clinical and nonclinical samples (α > 0.85)^84^. There is evidence of construct validity since the PSS-10 was found to correlate positively with depression, anxiety, fatigue, and procrastination and negatively with life satisfaction^85^.

#### Emotion regulation

Individual differences in the habitual engagement of cognitive reappraisal and expressive suppression were measured using the *Emotion Regulation Questionnaire* (ERQ^31,86^). The questionnaire contains 10 self-referential statements that relate to dealing with positive and negative feelings. The subscale reappraisal comprises 6 items and the subscale suppression four items, which are rated on a 7-point scale. The reappraisal score of the ERQ can range from 6 to 42, whereas the suppression score can range from 4 to 28. The internal consistency reported for expressive suppression (α = 0.74) and cognitive reappraisal (α = 0.76) of the German version of the ERQ can be considered sufficient^86^. There is evidence for factorial and concurrent validity of the ERQ^87^.

#### Alexithymia

Alexithymia was measured using the *20-Item Toronto Alexithymia Scale* (TAS-20^88^). The TAS-20 consists of the three subscales difficulties identifying feelings, difficulties describing feelings, and externally oriented thinking. The items are evaluated on a 5-point Likert scale. The total score of the TAS-20 can vary between 20 and 100. The internal consistency of the TAS-20 total score lies at 0.8 and can be evaluated as good^89^. For the TAS-20 evidence of construct validity has been provided in numerous studies^90,91^, which in general demonstrate that the total scale converges and diverges in theoretically meaningful ways with measures of closely related and unrelated constructs.

### Measure of intelligence

Intelligence was assessed by the *Mehrfachwahl-Wortschatz-Intelligenztest* version B (MWT-B^92^). The MWT-B is a performance test with no time restrictions that consists of 37 items. Each item comprises one real word, which has to be identified, and four pronounceable pseudowords. MWT-B sum scores can be converted into IQ scores. The MWT-B sum score shows high correlations with global IQ in healthy adults^93^.

### Free-viewing task

We administered a free-viewing task to measure attention allocation to emotional facial expressions. 140 faces of 20 models (10 male, 10 female) were selected from the Karolinska Directed Emotional Faces (KDEF^94^). For each model, a neutral expression and six emotional expressions were chosen (i.e., anger, disgust, fear, sadness, surprise, and happiness). In our viewing task, pairs of faces were displayed, an emotional face combined with a neutral face of the same model. All facial stimuli were presented in their original color on a grey background. Each trial started with a fixation cross at the center of the screen. Only when participants fixated on it for 1000 ms, the fixation cross was replaced with a face pair. Both faces were shown simultaneously for 5000 ms (positions of faces were counterbalanced). Immediately afterwards, the next trial was initiated with a fixation cross. Each of the six facial emotions was presented 20 times so that the viewing task consisted of 120 trials in total. Trials were shown in a fixed random order. Participants were instructed to freely view the pictures on the screen.

### Apparatus

The experimental design was realized using the PsychoPy software package v.2022.2.5^95^. For the presentation of experimental stimuli, a 23.8-inch wide-screen DELL monitor was used (resolution: 1920 × 1200). During the experiment, eye movements were continuously recorded with a Tobii Pro Fusion eye tracking system, which was fixed to the bottom of the monitor. The Tobii Pro Fusion eye tracker is able to compensate for head movements, therefore, no head-resting device was required. The laboratory was shielded from sunlight by closed roller blinds. Ceiling lighting produced a stable illuminance condition during the experiments. Study participants were seated approximately 40 cm from the monitor. The eye tracker was controlled by the experimental presentation software through the ioHub event monitoring framework. Data was recorded binocularly with a sampling rate of 120 Hz.

### General procedure

After a screening interview, participants who fulfilled the inclusion criteria and were still interested in partaking were invited to the eye tracking laboratory at the Department of Psychosomatic Medicine of the University of Leipzig. At the beginning of the individual experimental sessions, participants filled out the self-report questionnaires and tests under supervision of the experimenter and their visual acuity was tested with a Snellen eye chart. Hereafter, participants were seated in front of the eye-tracking monitor and were asked to find a comfortable position in which they could sit calmly throughout the task. All participants underwent the calibration procedure to guarantee adequate quality of the eye-tracking data. Finally, the free-viewing task was administered to participants.

### Statistical analysis

We based our analyses of the eye-tracking data on fixation duration as an index of late attention allocation. Specifically, total fixation duration was defined as the sum of all fixations on the emotional and neutral face respectively, which had a minimum duration of 100 ms. We calculated a total fixation duration bias score for each trial based on the difference between total fixation duration on the emotional face minus total fixation duration on the neutral face. A positive mean total fixation duration bias score indicates that on average total fixation duration on emotional faces was greater than that on neutral faces. To assess reliability of our attention measure, we calculated the Spearman-Brown corrected split-half reliability with use of the splithalf function from the splithalf package (version 0.8.2).

We used a repeated measure linear mixed model approach, using the lmer function from the lme4 (version 1.1–34) package to investigate the influence of traumatic experiences in childhood (using CTQ subscale scores) on late attention to emotional faces (total fixation duration bias scores). In our first linear model, we used the scores of the CTQ subscales, to investigate the effect of the different types of childhood maltreatment on attention allocation to faces of different emotional qualities, which represents our first research question. To achieve this, we included CTQ subscale scores as well as the remaining self-report and test variables (ERQ subscales, BDI-II, STAI, TAS-20, and PSS-10), position of the stimulus (left, right), and emotion (fear, anger, surprise, happiness, disgust, sadness) as fixed effects in our model. Furthermore, we integrated interaction effects between the CTQ subscales and the emotion condition, as we wanted to investigate the differential effect of childhood maltreatment subtypes on attention allocation for different emotional cues. We added a random intercept for our participants variable that accounts for both the initial differences between participants and the correlation of the repeated measures acquired by a subject. In our second research question, we examine whether the emotion regulation strategies of expressive suppression and cognitive reappraisal are related to late processes of attention allocation to facial emotions. The fixed effects of expressive suppression and cognitive reappraisal in our mixed linear model are used to test whether the emotion regulation strategies have an impact on attention to emotional information. We calculated a second linear model, which was identical to the first, except that we entered the CTQ total score instead of the CTQ subscale scores into the model. In this way, we investigated the effect of overall childhood maltreatment severity on attention allocation to faces of different emotional qualities.

Statistical analyses were performed in R (version 4.3.1) using RStudio (version 2023.06.0 + 421; RStudio, Inc.). Product-moment correlations were computed for self-report and test measures. Pairwise t-tests were carried out with the pairwise_t_test function from the rstatix (0.7.2) package in order to test the difference between total fixation duration on emotional versus neutral faces. Once the model was finalized, we entered it into a type 3 ANOVA with the Anova function from the car (version 3.1–2) package to receive the F statistic as basis of our hypothesis tests. The p-value was set at < 0.05. In case of significant main or interaction effects including a variable with more than two levels, namely emotion, we used the emmeans function from the emmeans (version 1.8.9) package to calculate estimated marginal means (EMM) and pairwise comparisons. EMMs were computed at four distinct points of every subscale, which represent distinct severity levels of maltreatment (none, low, moderate, and severe) (see^41^). In this way, we were able to contrast estimated marginal means between the emotion conditions and determine significant differences between them. Significance thresholds were adjusted for multiple comparisons using the Tukey method.

## Electronic supplementary material

Below is the link to the electronic supplementary material.

Supplementary Material 1

## Data Availability

The datasets used and/or analyzed during the current study are available from the corresponding author on reasonable request.
