# Supplementary material for: Attention to facial emotions in adult women varies by type and severity of childhood maltreatment experience and emotion regulation strategy
Source: Sci Rep. 2025 May 9;15:16266. doi: 10.1038/s41598-025-99562-z (PMC12064654; doi:10.1038/s41598-025-99562-z)
Supplement: Supplementary file 1 — Supplementary Material 1 [file 41598_2025_99562_MOESM1_ESM.docx]

Supplementary Information

***Attention to facial emotions in adult women varies by type and severity of childhood maltreatment experience and emotion regulation strategy***

Dennis Hoepfel, Anastasiia Bila, Vivien Günther, Anette Kersting and Thomas Suslow

#

**Table S1: Spearman-Brown and split-half reliability of fixation duration bias scores for all emotion conditions.**

| **Face conditions** | **Spearman-Brown**  **reliability** | **Split-half**  **reliability** |
| --- | --- | --- |
| Happiness - neutral | 0.93 | 0.88 |
| Surprise - neutral | 0.93 | 0.86 |
| Anger - neutral | 0.95 | 0.90 |
| Disgust - neutral | 0.96 | 0.92 |
| Fear - neutral | 0.95 | 0.90 |
| Sadness - neutral | 0.94 | 0.89 |

**Table S2. Comparison of fixation duration for emotional and paired neutral faces (*t*-test results).**

| **Face conditions** | **N1** | **N2** | ***t*** | **df** | ***p*** |
| --- | --- | --- | --- | --- | --- |
| Happiness - neutral | 2000 | 2000 | 24.96 | 1999 | <.001 |
| Surprise - neutral | 2000 | 2000 | 19.60 | 1999 | <.001 |
| Anger - neutral | 2000 | 2000 | 16.46 | 1999 | <.001 |
| Disgust - neutral | 2000 | 2000 | 14.16 | 1999 | <.001 |
| Fear - neutral | 2000 | 2000 | 16.54 | 1999 | <.001 |
| Sadness - neutral | 2000 | 2000 | 15.91 | 1999 | <.001 |

N1: Number of measurement values for fixation duration on specific emotional faces entered into the analysis.

N2: Number of measurement values for fixation duration on (paired) neutral faces entered into the analysis.

## Table S3: Estimated marginal means of total fixation duration bias for emotion conditions as a function of severity level of physical abuse.

| **Emotion** | **CTQ-PA** | **emmean** | **SE** | **df** | **lower CI** | **upper CI** |
| --- | --- | --- | --- | --- | --- | --- |
| fear-neutral | 6.0 | 668.4 | 98.19 | 116.3 | 473.894 | 862.8 |
| anger-neutral | 6.0 | 585.6 | 98.19 | 116.3 | 391.139 | 780.1 |
| disgust-neutral | 6.0 | 539.5 | 98.13 | 116.1 | 345.091 | 733.8 |
| happiness-neutral | 6.0 | 967.0 | 98.13 | 116.1 | 772.672 | 1161.4 |
| sadness-neutral | 6.0 | 617.5 | 98.13 | 116.1 | 423.101 | 811.8 |
| surprise-neutral | 6.0 | 730.1 | 98.26 | 116.7 | 535.535 | 924.7 |
| fear-neutral | 8.5 | 639.7 | 88.25 | 116.8 | 464.970 | 814.5 |
| anger-neutral | 8.5 | 666.9 | 88.25 | 116.8 | 492.102 | 841.6 |
| disgust-neutral | 8.5 | 590.9 | 88.19 | 116.4 | 416.227 | 765.5 |
| happiness-neutral | 8.5 | 976.5 | 88.19 | 116.4 | 801.877 | 1151.2 |
| sadness-neutral | 8.5 | 611.5 | 88.19 | 116.4 | 436.880 | 786.2 |
| surprise-neutral | 8.5 | 698.4 | 88.33 | 117.2 | 523.506 | 873.4 |
| fear-neutral | 11.0 | 611.1 | 117.41 | 115.8 | 378.575 | 843.7 |
| anger-neutral | 11.0 | 748.1 | 117.41 | 115.8 | 515.592 | 980.7 |
| disgust-neutral | 11.0 | 642.3 | 117.37 | 115.6 | 409.852 | 874.8 |
| happiness-neutral | 11.0 | 986.0 | 117.37 | 115.6 | 753.571 | 1218.5 |
| sadness-neutral | 11.0 | 605.6 | 117.37 | 115.6 | 373.146 | 838.1 |
| surprise-neutral | 11.0 | 666.7 | 117.48 | 116.1 | 434.061 | 899.4 |
| fear-neutral | 19.0 | 519.6 | 292.81 | 114.8 | -60.447 | 1099.6 |
| anger-neutral | 19.0 | 1008.2 | 292.81 | 114.8 | 428.205 | 1588.2 |
| disgust-neutral | 19.0 | 806.9 | 292.79 | 114.8 | 226.933 | 1386.9 |
| happiness-neutral | 19.0 | 1016.5 | 292.79 | 114.8 | 436.472 | 1596.4 |
| sadness-neutral | 19.0 | 586.7 | 292.79 | 114.8 | 6.682 | 1166.6 |
| surprise-neutral | 19.0 | 565.3 | 292.84 | 114.8 | -14.776 | 1145.3 |

CTQ-PA: physical abuse scale of the Childhood Trauma Questionnaire; emmean: estimated marginal mean of respective condition and CTQ subdimension severity level; SE: standard error; df: degrees of freedom; lower Cl: lower limit of the 95% confidence interval; upper CI: upper limit of the 95% confidence interval.

## Table S4: Contrasts between emotion conditions for severity levels of physical abuse.

| **Contrast** | **CTQ-PA** | **estimate** | **SE** | **df** | **t** | **p** |
| --- | --- | --- | --- | --- | --- | --- |
| (fear-neutral) - (anger-neutral) | 6.0 | 82.754 | 55.19 | 11869 | 1.4993 | 0.6647 |
| (fear-neutral) - (disgust-neutral) | 6.0 | 128.905 | 54.98 | 11869 | 2.3444 | 0.1765 |
| (fear-neutral) - (happiness-neutral) | 6.0 | -298.676 | 54.52 | 11869 | -5.4782 | 0.0000 |
| (fear-neutral) - (sadness-neutral) | 6.0 | 50.895 | 54.98 | 11869 | 0.9256 | 0.9400 |
| (fear-neutral) - (surprise-neutral) | 6.0 | -61.784 | 55.44 | 11869 | -1.1144 | 0.8756 |
| (anger-neutral) - (disgust-neutral) | 6.0 | 46.151 | 54.52 | 11869 | 0.8465 | 0.9587 |
| (anger-neutral) - (happiness-neutral) | 6.0 | -381.431 | 54.98 | 11869 | -6.9372 | 0.0000 |
| (anger-neutral) - (sadness-neutral) | 6.0 | -31.860 | 54.52 | 11869 | -0.5844 | 0.9921 |
| (anger-neutral) - (surprise-neutral) | 6.0 | -144.539 | 54.52 | 11869 | -2.6511 | 0.0855 |
| (disgust-neutral) - (happiness-neutral) | 6.0 | -427.582 | 54.81 | 11869 | -7.8011 | 0.0000 |
| (disgust-neutral) - (sadness-neutral) | 6.0 | -78.010 | 54.50 | 11869 | -1.4313 | 0.7079 |
| (disgust-neutral) - (surprise-neutral) | 6.0 | -190.690 | 54.58 | 11869 | -3.4938 | 0.0064 |
| (happiness-neutral) - (sadness-neutral) | 6.0 | 349.571 | 54.81 | 11869 | 6.3778 | 0.0000 |
| (happiness-neutral) - (surprise-neutral) | 6.0 | 236.892 | 55.19 | 11869 | 4.2920 | 0.0003 |
| (sadness-neutral) - (surprise-neutral) | 6.0 | -112.679 | 54.58 | 11869 | -2.0645 | 0.3061 |
| (fear-neutral) - (anger-neutral) | 8.5 | -27.131 | 49.96 | 11869 | -0.5431 | 0.9944 |
| (fear-neutral) - (disgust-neutral) | 8.5 | 48.857 | 49.73 | 11869 | 0.9825 | 0.9236 |
| (fear-neutral) - (happiness-neutral) | 8.5 | -336.793 | 49.21 | 11869 | -6.8436 | 0.0000 |
| (fear-neutral) - (sadness-neutral) | 8.5 | 28.204 | 49.73 | 11869 | 0.5672 | 0.9931 |
| (fear-neutral) - (surprise-neutral) | 8.5 | -58.695 | 50.23 | 11869 | -1.1685 | 0.8519 |
| (anger-neutral) - (disgust-neutral) | 8.5 | 75.988 | 49.21 | 11869 | 1.5441 | 0.6356 |
| (anger-neutral) - (happiness-neutral) | 8.5 | -309.662 | 49.73 | 11869 | -6.2275 | 0.0000 |
| (anger-neutral) - (sadness-neutral) | 8.5 | 55.336 | 49.21 | 11869 | 1.1244 | 0.8714 |
| (anger-neutral) - (surprise-neutral) | 8.5 | -31.563 | 49.21 | 11869 | -0.6414 | 0.9879 |
| (disgust-neutral) - (happiness-neutral) | 8.5 | -385.650 | 49.53 | 11869 | -7.7856 | 0.0000 |
| (disgust-neutral) - (sadness-neutral) | 8.5 | -20.653 | 49.19 | 11869 | -0.4198 | 0.9983 |
| (disgust-neutral) - (surprise-neutral) | 8.5 | -107.552 | 49.28 | 11869 | -2.1826 | 0.2459 |
| (happiness-neutral) - (sadness-neutral) | 8.5 | 364.998 | 49.53 | 11869 | 7.3687 | 0.0000 |
| (happiness-neutral) - (surprise-neutral) | 8.5 | 278.099 | 49.96 | 11869 | 5.5667 | 0.0000 |
| (sadness-neutral) - (surprise-neutral) | 8.5 | -86.899 | 49.28 | 11869 | -1.7635 | 0.4896 |
| (fear-neutral) - (anger-neutral) | 11.0 | -137.017 | 65.41 | 11869 | -2.0948 | 0.2899 |
| (fear-neutral) - (disgust-neutral) | 11.0 | -31.191 | 65.23 | 11869 | -0.4782 | 0.9969 |
| (fear-neutral) - (happiness-neutral) | 11.0 | -374.911 | 64.84 | 11869 | -5.7820 | 0.0000 |
| (fear-neutral) - (sadness-neutral) | 11.0 | 5.514 | 65.23 | 11869 | 0.0845 | 1.0000 |
| (fear-neutral) - (surprise-neutral) | 11.0 | -55.605 | 65.62 | 11869 | -0.8474 | 0.9585 |
| (anger-neutral) - (disgust-neutral) | 11.0 | 105.826 | 64.84 | 11869 | 1.6321 | 0.5771 |
| (anger-neutral) - (happiness-neutral) | 11.0 | -237.893 | 65.23 | 11869 | -3.6470 | 0.0036 |
| (anger-neutral) - (sadness-neutral) | 11.0 | 142.531 | 64.84 | 11869 | 2.1982 | 0.2385 |
| (anger-neutral) - (surprise-neutral) | 11.0 | 81.412 | 64.84 | 11869 | 1.2556 | 0.8091 |
| (disgust-neutral) - (happiness-neutral) | 11.0 | -343.719 | 65.08 | 11869 | -5.2811 | 0.0000 |
| (disgust-neutral) - (sadness-neutral) | 11.0 | 36.705 | 64.82 | 11869 | 0.5662 | 0.9932 |
| (disgust-neutral) - (surprise-neutral) | 11.0 | -24.414 | 64.89 | 11869 | -0.3762 | 0.9990 |
| (happiness-neutral) - (sadness-neutral) | 11.0 | 380.424 | 65.08 | 11869 | 5.8451 | 0.0000 |
| (happiness-neutral) - (surprise-neutral) | 11.0 | 319.305 | 65.41 | 11869 | 4.8817 | 0.0000 |
| (sadness-neutral) - (surprise-neutral) | 11.0 | -61.119 | 64.89 | 11869 | -0.9419 | 0.9356 |
| (fear-neutral) - (anger-neutral) | 19.0 | -488.652 | 160.19 | 11869 | -3.0505 | 0.0277 |
| (fear-neutral) - (disgust-neutral) | 19.0 | -287.346 | 160.11 | 11869 | -1.7946 | 0.4692 |
| (fear-neutral) - (happiness-neutral) | 19.0 | -496.885 | 159.96 | 11869 | -3.1064 | 0.0233 |
| (fear-neutral) - (sadness-neutral) | 19.0 | -67.095 | 160.11 | 11869 | -0.4190 | 0.9984 |
| (fear-neutral) - (surprise-neutral) | 19.0 | -45.719 | 160.27 | 11869 | -0.2853 | 0.9997 |
| (anger-neutral) - (disgust-neutral) | 19.0 | 201.307 | 159.96 | 11869 | 1.2585 | 0.8076 |
| (anger-neutral) - (happiness-neutral) | 19.0 | -8.233 | 160.11 | 11869 | -0.0514 | 1.0000 |
| (anger-neutral) - (sadness-neutral) | 19.0 | 421.557 | 159.96 | 11869 | 2.6355 | 0.0889 |
| (anger-neutral) - (surprise-neutral) | 19.0 | 442.933 | 159.96 | 11869 | 2.7691 | 0.0626 |
| (disgust-neutral) - (happiness-neutral) | 19.0 | -209.540 | 160.05 | 11869 | -1.3092 | 0.7801 |
| (disgust-neutral) - (sadness-neutral) | 19.0 | 220.250 | 159.95 | 11869 | 1.3770 | 0.7410 |
| (disgust-neutral) - (surprise-neutral) | 19.0 | 241.627 | 159.98 | 11869 | 1.5104 | 0.6576 |
| (happiness-neutral) - (sadness-neutral) | 19.0 | 429.790 | 160.05 | 11869 | 2.6853 | 0.0782 |
| (happiness-neutral) - (surprise-neutral) | 19.0 | 451.166 | 160.19 | 11869 | 2.8165 | 0.0549 |
| (sadness-neutral) - (surprise-neutral) | 19.0 | 21.377 | 159.98 | 11869 | 0.1336 | 1.0000 |

CTQ-PA: physical abuse scale of the Childhood Trauma Questionnaire; Estimate: difference of estimated marginal means (in milliseconds) between the contrasted conditions; SE: standard error; df: degrees of freedom; t: t statistic.

## Table S5: Estimated marginal means of total fixation duration bias for emotion conditions as a function of severity level of emotional abuse.

| **Emotion** | **CTQ-EA** | **emmean** | **SE** | **df** | **lower CI** | **upper CI** |
| --- | --- | --- | --- | --- | --- | --- |
| fear-neutral | 6.5 | 703.1 | 238.06 | 114.9 | 231.543 | 1174.7 |
| anger-neutral | 6.5 | 709.4 | 238.06 | 114.9 | 237.870 | 1181.0 |
| disgust-neutral | 6.5 | 832.5 | 238.04 | 114.9 | 361.010 | 1304.0 |
| happiness-neutral | 6.5 | 1142.6 | 238.04 | 114.9 | 671.110 | 1614.1 |
| sadness-neutral | 6.5 | 461.8 | 238.04 | 114.9 | -9.721 | 933.3 |
| surprise-neutral | 6.5 | 677.3 | 238.09 | 115.0 | 205.681 | 1148.9 |
| fear-neutral | 10.5 | 678.6 | 151.68 | 115.3 | 378.180 | 979.1 |
| anger-neutral | 10.5 | 681.4 | 151.68 | 115.3 | 380.909 | 981.8 |
| disgust-neutral | 10.5 | 720.6 | 151.65 | 115.2 | 420.209 | 1021.0 |
| happiness-neutral | 10.5 | 1068.7 | 151.65 | 115.2 | 768.300 | 1369.1 |
| sadness-neutral | 10.5 | 528.2 | 151.65 | 115.2 | 227.779 | 828.5 |
| surprise-neutral | 10.5 | 690.2 | 151.73 | 115.5 | 389.703 | 990.8 |
| fear-neutral | 14.0 | 657.2 | 95.21 | 116.5 | 468.640 | 845.8 |
| anger-neutral | 14.0 | 656.8 | 95.21 | 116.5 | 468.220 | 845.4 |
| disgust-neutral | 14.0 | 622.6 | 95.16 | 116.2 | 434.178 | 811.1 |
| happiness-neutral | 14.0 | 1004.0 | 95.16 | 116.2 | 815.512 | 1192.4 |
| sadness-neutral | 14.0 | 586.2 | 95.16 | 116.2 | 397.762 | 774.7 |
| surprise-neutral | 14.0 | 701.6 | 95.29 | 116.8 | 512.849 | 890.3 |
| fear-neutral | 20.5 | 617.4 | 146.93 | 115.4 | 326.401 | 908.5 |
| anger-neutral | 20.5 | 611.2 | 146.93 | 115.4 | 320.131 | 902.2 |
| disgust-neutral | 20.5 | 440.7 | 146.90 | 115.3 | 149.775 | 731.7 |
| happiness-neutral | 20.5 | 883.8 | 146.90 | 115.3 | 592.844 | 1174.8 |
| sadness-neutral | 20.5 | 694.1 | 146.90 | 115.3 | 403.097 | 985.0 |
| surprise-neutral | 20.5 | 722.6 | 146.98 | 115.5 | 431.466 | 1013.7 |

CTQ-EA: emotional abuse scale of the Childhood Trauma Questionnaire; emmean: estimated marginal mean of respective condition and CTQ subdimension severity level; SE: standard error; df: degrees of freedom; lower Cl: lower limit of the 95% confidence interval; upper CI: upper limit of the 95% confidence interval.

## Table S6: Contrasts between emotion conditions for severity levels of emotional abuse.

| **Contrast** | **CTQ-EA** | **estimate** | **SE** | **df** | **t** | **p** |
| --- | --- | --- | --- | --- | --- | --- |
| (fear-neutral) - (anger-neutral) | 6.5 | -6.3274 | 130.47 | 11869 | -0.0485 | 1.0000 |
| (fear-neutral) - (disgust-neutral) | 6.5 | -129.4249 | 130.38 | 11869 | -0.9927 | 0.9205 |
| (fear-neutral) - (happiness-neutral) | 6.5 | -439.5253 | 130.19 | 11869 | -3.3761 | 0.0096 |
| (fear-neutral) - (sadness-neutral) | 6.5 | 241.3062 | 130.38 | 11869 | 1.8508 | 0.4330 |
| (fear-neutral) - (surprise-neutral) | 6.5 | 25.8026 | 130.58 | 11869 | 0.1976 | 1.0000 |
| (anger-neutral) - (disgust-neutral) | 6.5 | -123.0975 | 130.19 | 11869 | -0.9455 | 0.9346 |
| (anger-neutral) - (happiness-neutral) | 6.5 | -433.1979 | 130.38 | 11869 | -3.3225 | 0.0115 |
| (anger-neutral) - (sadness-neutral) | 6.5 | 247.6336 | 130.19 | 11869 | 1.9021 | 0.4008 |
| (anger-neutral) - (surprise-neutral) | 6.5 | 32.1300 | 130.19 | 11869 | 0.2468 | 0.9999 |
| (disgust-neutral) - (happiness-neutral) | 6.5 | -310.1004 | 130.31 | 11869 | -2.3797 | 0.1634 |
| (disgust-neutral) - (sadness-neutral) | 6.5 | 370.7311 | 130.18 | 11869 | 2.8478 | 0.0503 |
| (disgust-neutral) - (surprise-neutral) | 6.5 | 155.2275 | 130.21 | 11869 | 1.1921 | 0.8409 |
| (happiness-neutral) - (sadness-neutral) | 6.5 | 680.8315 | 130.31 | 11869 | 5.2247 | 0.0000 |
| (happiness-neutral) - (surprise-neutral) | 6.5 | 465.3278 | 130.47 | 11869 | 3.5665 | 0.0049 |
| (sadness-neutral) - (surprise-neutral) | 6.5 | -215.5036 | 130.21 | 11869 | -1.6550 | 0.5618 |
| (fear-neutral) - (anger-neutral) | 10.5 | -2.7283 | 83.78 | 11869 | -0.0326 | 1.0000 |
| (fear-neutral) - (disgust-neutral) | 10.5 | -41.9624 | 83.64 | 11869 | -0.5017 | 0.9961 |
| (fear-neutral) - (happiness-neutral) | 10.5 | -390.0539 | 83.34 | 11869 | -4.6805 | 0.0000 |
| (fear-neutral) - (sadness-neutral) | 10.5 | 150.4678 | 83.64 | 11869 | 1.7990 | 0.4664 |
| (fear-neutral) - (surprise-neutral) | 10.5 | -11.6154 | 83.94 | 11869 | -0.1384 | 1.0000 |
| (anger-neutral) - (disgust-neutral) | 10.5 | -39.2340 | 83.34 | 11869 | -0.4708 | 0.9971 |
| (anger-neutral) - (happiness-neutral) | 10.5 | -387.3256 | 83.64 | 11869 | -4.6309 | 0.0001 |
| (anger-neutral) - (sadness-neutral) | 10.5 | 153.1961 | 83.34 | 11869 | 1.8383 | 0.4410 |
| (anger-neutral) - (surprise-neutral) | 10.5 | -8.8871 | 83.34 | 11869 | -0.1066 | 1.0000 |
| (disgust-neutral) - (happiness-neutral) | 10.5 | -348.0915 | 83.53 | 11869 | -4.1675 | 0.0004 |
| (disgust-neutral) - (sadness-neutral) | 10.5 | 192.4301 | 83.32 | 11869 | 2.3095 | 0.1902 |
| (disgust-neutral) - (surprise-neutral) | 10.5 | 30.3470 | 83.37 | 11869 | 0.3640 | 0.9992 |
| (happiness-neutral) - (sadness-neutral) | 10.5 | 540.5217 | 83.53 | 11869 | 6.4714 | 0.0000 |
| (happiness-neutral) - (surprise-neutral) | 10.5 | 378.4385 | 83.78 | 11869 | 4.5172 | 0.0001 |
| (sadness-neutral) - (surprise-neutral) | 10.5 | -162.0832 | 83.37 | 11869 | -1.9441 | 0.3752 |
| (fear-neutral) - (anger-neutral) | 14.0 | 0.4209 | 53.62 | 11869 | 0.0078 | 1.0000 |
| (fear-neutral) - (disgust-neutral) | 14.0 | 34.5674 | 53.41 | 11869 | 0.6473 | 0.9874 |
| (fear-neutral) - (happiness-neutral) | 14.0 | -346.7664 | 52.93 | 11869 | -6.5514 | 0.0000 |
| (fear-neutral) - (sadness-neutral) | 14.0 | 70.9842 | 53.41 | 11869 | 1.3291 | 0.7689 |
| (fear-neutral) - (surprise-neutral) | 14.0 | -44.3561 | 53.88 | 11869 | -0.8233 | 0.9633 |
| (anger-neutral) - (disgust-neutral) | 14.0 | 34.1465 | 52.93 | 11869 | 0.6451 | 0.9875 |
| (anger-neutral) - (happiness-neutral) | 14.0 | -347.1873 | 53.41 | 11869 | -6.5009 | 0.0000 |
| (anger-neutral) - (sadness-neutral) | 14.0 | 70.5633 | 52.93 | 11869 | 1.3331 | 0.7666 |
| (anger-neutral) - (surprise-neutral) | 14.0 | -44.7770 | 52.93 | 11869 | -0.8460 | 0.9588 |
| (disgust-neutral) - (happiness-neutral) | 14.0 | -381.3338 | 53.23 | 11869 | -7.1642 | 0.0000 |
| (disgust-neutral) - (sadness-neutral) | 14.0 | 36.4168 | 52.91 | 11869 | 0.6883 | 0.9833 |
| (disgust-neutral) - (surprise-neutral) | 14.0 | -78.9235 | 52.99 | 11869 | -1.4894 | 0.6711 |
| (happiness-neutral) - (sadness-neutral) | 14.0 | 417.7506 | 53.23 | 11869 | 7.8483 | 0.0000 |
| (happiness-neutral) - (surprise-neutral) | 14.0 | 302.4103 | 53.62 | 11869 | 5.6396 | 0.0000 |
| (sadness-neutral) - (surprise-neutral) | 14.0 | -115.3403 | 52.99 | 11869 | -2.1767 | 0.2487 |
| (fear-neutral) - (anger-neutral) | 20.5 | 6.2694 | 81.22 | 11869 | 0.0772 | 1.0000 |
| (fear-neutral) - (disgust-neutral) | 20.5 | 176.6940 | 81.08 | 11869 | 2.1793 | 0.2475 |
| (fear-neutral) - (happiness-neutral) | 20.5 | -266.3754 | 80.77 | 11869 | -3.2981 | 0.0125 |
| (fear-neutral) - (sadness-neutral) | 20.5 | -76.6282 | 81.08 | 11869 | -0.9451 | 0.9347 |
| (fear-neutral) - (surprise-neutral) | 20.5 | -105.1603 | 81.39 | 11869 | -1.2920 | 0.7896 |
| (anger-neutral) - (disgust-neutral) | 20.5 | 170.4247 | 80.77 | 11869 | 2.1101 | 0.2820 |
| (anger-neutral) - (happiness-neutral) | 20.5 | -272.6447 | 81.08 | 11869 | -3.3627 | 0.0101 |
| (anger-neutral) - (sadness-neutral) | 20.5 | -82.8976 | 80.77 | 11869 | -1.0264 | 0.9092 |
| (anger-neutral) - (surprise-neutral) | 20.5 | -111.4297 | 80.77 | 11869 | -1.3796 | 0.7394 |
| (disgust-neutral) - (happiness-neutral) | 20.5 | -443.0694 | 80.96 | 11869 | -5.4725 | 0.0000 |
| (disgust-neutral) - (sadness-neutral) | 20.5 | -253.3222 | 80.75 | 11869 | -3.1370 | 0.0212 |
| (disgust-neutral) - (surprise-neutral) | 20.5 | -281.8543 | 80.81 | 11869 | -3.4880 | 0.0065 |
| (happiness-neutral) - (sadness-neutral) | 20.5 | 189.7472 | 80.96 | 11869 | 2.3436 | 0.1768 |
| (happiness-neutral) - (surprise-neutral) | 20.5 | 161.2151 | 81.22 | 11869 | 1.9849 | 0.3510 |
| (sadness-neutral) - (surprise-neutral) | 20.5 | -28.5321 | 80.81 | 11869 | -0.3531 | 0.9993 |

CTQ-EA: emotional abuse scale of the Childhood Trauma Questionnaire; Estimate: difference of estimated marginal means (in milliseconds) between the contrasted conditions; SE: standard error; df: degrees of freedom; t: t statistic.

## Table S7: Estimated marginal means of total fixation duration bias for emotion conditions as a function of severity level of emotional neglect.

| **Emotion** | **CTQ-EN** | **emmean** | **SE** | **df** | **lower CI** | **upper CI** |
| --- | --- | --- | --- | --- | --- | --- |
| fear-neutral | 7.0 | 1099.4 | 318.5 | 115.2 | 468.5 | 1730.3 |
| anger-neutral | 7.0 | 863.5 | 318.5 | 115.2 | 232.5 | 1494.4 |
| disgust-neutral | 7.0 | 785.7 | 318.5 | 115.2 | 154.8 | 1416.6 |
| happiness-neutral | 7.0 | 1023.7 | 318.5 | 115.2 | 392.8 | 1654.6 |
| sadness-neutral | 7.0 | 1162.7 | 318.5 | 115.2 | 531.8 | 1793.6 |
| surprise-neutral | 7.0 | 922.5 | 318.5 | 115.2 | 291.5 | 1553.4 |
| fear-neutral | 12.0 | 897.2 | 190.3 | 115.4 | 520.3 | 1274.2 |
| anger-neutral | 12.0 | 765.9 | 190.3 | 115.4 | 389.0 | 1142.9 |
| disgust-neutral | 12.0 | 692.5 | 190.3 | 115.4 | 315.6 | 1069.4 |
| happiness-neutral | 12.0 | 1001.5 | 190.3 | 115.4 | 624.6 | 1378.4 |
| sadness-neutral | 12.0 | 916.9 | 190.3 | 115.4 | 540.0 | 1293.8 |
| surprise-neutral | 12.0 | 826.0 | 190.4 | 115.5 | 449.0 | 1203.1 |
| fear-neutral | 16.0 | 735.5 | 105.3 | 116.3 | 526.9 | 944.0 |
| anger-neutral | 16.0 | 687.9 | 105.3 | 116.3 | 479.4 | 896.5 |
| disgust-neutral | 16.0 | 617.9 | 105.2 | 116.0 | 409.5 | 826.4 |
| happiness-neutral | 16.0 | 983.7 | 105.2 | 116.0 | 775.3 | 1192.2 |
| sadness-neutral | 16.0 | 720.3 | 105.2 | 116.0 | 511.8 | 928.7 |
| surprise-neutral | 16.0 | 748.9 | 105.4 | 116.6 | 540.2 | 957.5 |
| fear-neutral | 21.5 | 513.0 | 125.7 | 115.9 | 264.1 | 762.0 |
| anger-neutral | 21.5 | 580.7 | 125.7 | 115.9 | 331.7 | 829.6 |
| disgust-neutral | 21.5 | 515.4 | 125.6 | 115.7 | 266.5 | 764.3 |
| happiness-neutral | 21.5 | 959.3 | 125.6 | 115.7 | 710.4 | 1208.1 |
| sadness-neutral | 21.5 | 449.9 | 125.6 | 115.7 | 201.0 | 698.8 |
| surprise-neutral | 21.5 | 642.8 | 125.8 | 116.1 | 393.7 | 891.8 |

CTQ-EN: emotional neglect scale of the Childhood Trauma Questionnaire; emmean: estimated marginal mean of respective condition and CTQ subdimension severity level; SE: standard error; df: degrees of freedom; lower Cl: lower limit of the 95% confidence interval; upper CI: upper limit of the 95% confidence interval.

## Table S8: Contrasts between emotion conditions for severity levels of emotional neglect.

| **Contrast** | **CTQ-EN** | **estimate** | **SE** | **df** | **t** | **p** |
| --- | --- | --- | --- | --- | --- | --- |
| (fear-neutral) - (anger-neutral) | 7.0 | 235.948 | 175.25 | 11869 | 1.3463 | 0.7590 |
| (fear-neutral) - (disgust-neutral) | 7.0 | 313.700 | 175.18 | 11869 | 1.7907 | 0.4718 |
| (fear-neutral) - (happiness-neutral) | 7.0 | 75.670 | 175.04 | 11869 | 0.4323 | 0.9981 |
| (fear-neutral) - (sadness-neutral) | 7.0 | -63.257 | 175.18 | 11869 | -0.3611 | 0.9992 |
| (fear-neutral) - (surprise-neutral) | 7.0 | 176.947 | 175.33 | 11869 | 1.0092 | 0.9151 |
| (anger-neutral) - (disgust-neutral) | 7.0 | 77.752 | 175.04 | 11869 | 0.4442 | 0.9978 |
| (anger-neutral) - (happiness-neutral) | 7.0 | -160.278 | 175.18 | 11869 | -0.9149 | 0.9428 |
| (anger-neutral) - (sadness-neutral) | 7.0 | -299.205 | 175.04 | 11869 | -1.7093 | 0.5255 |
| (anger-neutral) - (surprise-neutral) | 7.0 | -59.001 | 175.04 | 11869 | -0.3371 | 0.9994 |
| (disgust-neutral) - (happiness-neutral) | 7.0 | -238.030 | 175.13 | 11869 | -1.3592 | 0.7515 |
| (disgust-neutral) - (sadness-neutral) | 7.0 | -376.957 | 175.03 | 11869 | -2.1536 | 0.2599 |
| (disgust-neutral) - (surprise-neutral) | 7.0 | -136.754 | 175.06 | 11869 | -0.7812 | 0.9708 |
| (happiness-neutral) - (sadness-neutral) | 7.0 | -138.927 | 175.13 | 11869 | -0.7933 | 0.9687 |
| (happiness-neutral) - (surprise-neutral) | 7.0 | 101.276 | 175.25 | 11869 | 0.5779 | 0.9925 |
| (sadness-neutral) - (surprise-neutral) | 7.0 | 240.203 | 175.06 | 11869 | 1.3721 | 0.7438 |
| (fear-neutral) - (anger-neutral) | 12.0 | 131.266 | 105.17 | 11869 | 1.2481 | 0.8130 |
| (fear-neutral) - (disgust-neutral) | 12.0 | 204.706 | 105.06 | 11869 | 1.9484 | 0.3726 |
| (fear-neutral) - (happiness-neutral) | 12.0 | -104.294 | 104.82 | 11869 | -0.9950 | 0.9197 |
| (fear-neutral) - (sadness-neutral) | 12.0 | -19.674 | 105.06 | 11869 | -0.1873 | 1.0000 |
| (fear-neutral) - (surprise-neutral) | 12.0 | 71.197 | 105.30 | 11869 | 0.6761 | 0.9846 |
| (anger-neutral) - (disgust-neutral) | 12.0 | 73.441 | 104.82 | 11869 | 0.7006 | 0.9819 |
| (anger-neutral) - (happiness-neutral) | 12.0 | -235.560 | 105.06 | 11869 | -2.2421 | 0.2186 |
| (anger-neutral) - (sadness-neutral) | 12.0 | -150.940 | 104.82 | 11869 | -1.4400 | 0.7025 |
| (anger-neutral) - (surprise-neutral) | 12.0 | -60.069 | 104.82 | 11869 | -0.5731 | 0.9928 |
| (disgust-neutral) - (happiness-neutral) | 12.0 | -309.001 | 104.97 | 11869 | -2.9437 | 0.0382 |
| (disgust-neutral) - (sadness-neutral) | 12.0 | -224.380 | 104.81 | 11869 | -2.1408 | 0.2663 |
| (disgust-neutral) - (surprise-neutral) | 12.0 | -133.510 | 104.85 | 11869 | -1.2733 | 0.7997 |
| (happiness-neutral) - (sadness-neutral) | 12.0 | 84.621 | 104.97 | 11869 | 0.8061 | 0.9665 |
| (happiness-neutral) - (surprise-neutral) | 12.0 | 175.491 | 105.17 | 11869 | 1.6686 | 0.5527 |
| (sadness-neutral) - (surprise-neutral) | 12.0 | 90.870 | 104.85 | 11869 | 0.8667 | 0.9544 |
| (fear-neutral) - (anger-neutral) | 16.0 | 47.520 | 59.08 | 11869 | 0.8043 | 0.9668 |
| (fear-neutral) - (disgust-neutral) | 16.0 | 117.511 | 58.88 | 11869 | 1.9957 | 0.3447 |
| (fear-neutral) - (happiness-neutral) | 16.0 | -248.266 | 58.45 | 11869 | -4.2474 | 0.0003 |
| (fear-neutral) - (sadness-neutral) | 16.0 | 15.193 | 58.88 | 11869 | 0.2580 | 0.9998 |
| (fear-neutral) - (surprise-neutral) | 16.0 | -13.403 | 59.31 | 11869 | -0.2260 | 0.9999 |
| (anger-neutral) - (disgust-neutral) | 16.0 | 69.991 | 58.45 | 11869 | 1.1974 | 0.8383 |
| (anger-neutral) - (happiness-neutral) | 16.0 | -295.786 | 58.88 | 11869 | -5.0233 | 0.0000 |
| (anger-neutral) - (sadness-neutral) | 16.0 | -32.328 | 58.45 | 11869 | -0.5531 | 0.9939 |
| (anger-neutral) - (surprise-neutral) | 16.0 | -60.923 | 58.45 | 11869 | -1.0423 | 0.9036 |
| (disgust-neutral) - (happiness-neutral) | 16.0 | -365.777 | 58.72 | 11869 | -6.2290 | 0.0000 |
| (disgust-neutral) - (sadness-neutral) | 16.0 | -102.319 | 58.43 | 11869 | -1.7510 | 0.4978 |
| (disgust-neutral) - (surprise-neutral) | 16.0 | -130.915 | 58.51 | 11869 | -2.2376 | 0.2205 |
| (happiness-neutral) - (sadness-neutral) | 16.0 | 263.458 | 58.72 | 11869 | 4.4866 | 0.0001 |
| (happiness-neutral) - (surprise-neutral) | 16.0 | 234.863 | 59.08 | 11869 | 3.9753 | 0.0010 |
| (sadness-neutral) - (surprise-neutral) | 16.0 | -28.596 | 58.51 | 11869 | -0.4888 | 0.9966 |
| (fear-neutral) - (anger-neutral) | 21.5 | -67.630 | 70.07 | 11869 | -0.9652 | 0.9289 |
| (fear-neutral) - (disgust-neutral) | 21.5 | -2.382 | 69.91 | 11869 | -0.0341 | 1.0000 |
| (fear-neutral) - (happiness-neutral) | 21.5 | -446.227 | 69.54 | 11869 | -6.4166 | 0.0000 |
| (fear-neutral) - (sadness-neutral) | 21.5 | 63.134 | 69.91 | 11869 | 0.9031 | 0.9458 |
| (fear-neutral) - (surprise-neutral) | 21.5 | -129.728 | 70.27 | 11869 | -1.8462 | 0.4359 |
| (anger-neutral) - (disgust-neutral) | 21.5 | 65.248 | 69.54 | 11869 | 0.9383 | 0.9366 |
| (anger-neutral) - (happiness-neutral) | 21.5 | -378.596 | 69.91 | 11869 | -5.4158 | 0.0000 |
| (anger-neutral) - (sadness-neutral) | 21.5 | 130.764 | 69.54 | 11869 | 1.8804 | 0.4143 |
| (anger-neutral) - (surprise-neutral) | 21.5 | -62.098 | 69.54 | 11869 | -0.8930 | 0.9483 |
| (disgust-neutral) - (happiness-neutral) | 21.5 | -443.845 | 69.77 | 11869 | -6.3616 | 0.0000 |
| (disgust-neutral) - (sadness-neutral) | 21.5 | 65.516 | 69.53 | 11869 | 0.9423 | 0.9355 |
| (disgust-neutral) - (surprise-neutral) | 21.5 | -127.346 | 69.59 | 11869 | -1.8300 | 0.4463 |
| (happiness-neutral) - (sadness-neutral) | 21.5 | 509.361 | 69.77 | 11869 | 7.3006 | 0.0000 |
| (happiness-neutral) - (surprise-neutral) | 21.5 | 316.499 | 70.07 | 11869 | 4.5168 | 0.0001 |
| (sadness-neutral) - (surprise-neutral) | 21.5 | -192.862 | 69.59 | 11869 | -2.7715 | 0.0622 |

CTQ-EN: emotional neglect scale of the Childhood Trauma Questionnaire; Estimate: difference of estimated marginal means (in milliseconds) between the contrasted conditions; SE: standard error; df: degrees of freedom; t: t statistic.

## Table S9: Estimated marginal means of total fixation duration bias for emotion conditions as a function of level of overall childhood maltreatment severity.

| **Emotion** | **CTQ** | **emmean** | **SE** | **df** | **lower CI** | **upper CI** |
| --- | --- | --- | --- | --- | --- | --- |
| fear-neutral | 30.5 | 749.3 | 238.16 | Inf | 282.494 | 1216.1 |
| anger-neutral | 30.5 | 224.5 | 238.16 | Inf | -242.294 | 691.3 |
| disgust-neutral | 30.5 | 449.1 | 238.14 | Inf | -17.656 | 915.8 |
| happiness-neutral | 30.5 | 1226.9 | 238.14 | Inf | 760.189 | 1693.7 |
| sadness-neutral | 30.5 | 527.0 | 238.14 | Inf | 60.270 | 993.8 |
| surprise-neutral | 30.5 | 634.4 | 238.19 | Inf | 167.597 | 1101.3 |
| fear-neutral | 43.5 | 701.6 | 146.41 | Inf | 414.634 | 988.5 |
| anger-neutral | 43.5 | 421.3 | 146.41 | Inf | 134.372 | 708.3 |
| disgust-neutral | 43.5 | 509.0 | 146.37 | Inf | 222.161 | 795.9 |
| happiness-neutral | 43.5 | 1108.7 | 146.37 | Inf | 821.782 | 1395.6 |
| sadness-neutral | 43.5 | 567.3 | 146.37 | Inf | 280.386 | 854.2 |
| surprise-neutral | 43.5 | 668.3 | 146.46 | Inf | 381.228 | 955.3 |
| fear-neutral | 59.5 | 642.9 | 87.01 | Inf | 472.352 | 813.4 |
| anger-neutral | 59.5 | 663.6 | 87.01 | Inf | 493.045 | 834.1 |
| disgust-neutral | 59.5 | 582.8 | 86.95 | Inf | 412.419 | 753.3 |
| happiness-neutral | 59.5 | 963.1 | 86.95 | Inf | 792.688 | 1133.5 |
| sadness-neutral | 59.5 | 616.8 | 86.95 | Inf | 446.395 | 787.2 |
| surprise-neutral | 59.5 | 709.9 | 87.10 | Inf | 539.221 | 880.6 |
| fear-neutral | 96.5 | 507.2 | 316.94 | Inf | -114.039 | 1128.4 |
| anger-neutral | 96.5 | 1223.8 | 316.94 | Inf | 602.613 | 1845.0 |
| disgust-neutral | 96.5 | 753.5 | 316.93 | Inf | 132.328 | 1374.7 |
| happiness-neutral | 96.5 | 626.5 | 316.93 | Inf | 5.345 | 1247.7 |
| sadness-neutral | 96.5 | 731.4 | 316.93 | Inf | 110.231 | 1352.6 |
| surprise-neutral | 96.5 | 806.2 | 316.97 | Inf | 184.995 | 1427.5 |

##

CTQ: total score of the Childhood Trauma Questionnaire; emmean: estimated marginal mean of respective condition and CTQ severity level; SE: standard error; df: degrees of freedom; lower Cl: lower limit of the 95% confidence interval; upper CI: upper limit of the 95% confidence interval.

## Table S10: Contrasts between emotion conditions for severity levels of overall childhood maltreatment.

| **Contrast** | **CTQ** | **estimate** | **SE** | **df** | **t** | **p** |
| --- | --- | --- | --- | --- | --- | --- |
| (fear-neutral) - (anger-neutral) | 30.5 | 524.79 | 126.72 | Inf | 4.1413 | 0.0005 |
| (fear-neutral) - (disgust-neutral) | 30.5 | 300.19 | 126.63 | Inf | 2.3706 | 0.1666 |
| (fear-neutral) - (happiness-neutral) | 30.5 | -477.65 | 126.43 | Inf | -3.7780 | 0.0022 |
| (fear-neutral) - (sadness-neutral) | 30.5 | 222.27 | 126.63 | Inf | 1.7553 | 0.4950 |
| (fear-neutral) - (surprise-neutral) | 30.5 | 114.84 | 126.83 | Inf | 0.9054 | 0.9453 |
| (anger-neutral) - (disgust-neutral) | 30.5 | -224.59 | 126.43 | Inf | -1.7764 | 0.4811 |
| (anger-neutral) - (happiness-neutral) | 30.5 | -1002.44 | 126.63 | Inf | -7.9163 | 0.0000 |
| (anger-neutral) - (sadness-neutral) | 30.5 | -302.52 | 126.43 | Inf | -2.3928 | 0.1586 |
| (anger-neutral) - (surprise-neutral) | 30.5 | -409.95 | 126.43 | Inf | -3.2426 | 0.0150 |
| (disgust-neutral) - (happiness-neutral) | 30.5 | -777.84 | 126.55 | Inf | -6.1463 | 0.0000 |
| (disgust-neutral) - (sadness-neutral) | 30.5 | -77.93 | 126.42 | Inf | -0.6164 | 0.9899 |
| (disgust-neutral) - (surprise-neutral) | 30.5 | -185.36 | 126.45 | Inf | -1.4658 | 0.6862 |
| (happiness-neutral) - (sadness-neutral) | 30.5 | 699.92 | 126.55 | Inf | 5.5306 | 0.0000 |
| (happiness-neutral) - (surprise-neutral) | 30.5 | 592.49 | 126.72 | Inf | 4.6755 | 0.0000 |
| (sadness-neutral) - (surprise-neutral) | 30.5 | -107.43 | 126.45 | Inf | -0.8496 | 0.9581 |
| (fear-neutral) - (anger-neutral) | 43.5 | 280.26 | 79.30 | Inf | 3.5341 | 0.0055 |
| (fear-neutral) - (disgust-neutral) | 43.5 | 192.54 | 79.16 | Inf | 2.4325 | 0.1451 |
| (fear-neutral) - (happiness-neutral) | 43.5 | -407.08 | 78.83 | Inf | -5.1638 | 0.0000 |
| (fear-neutral) - (sadness-neutral) | 43.5 | 134.32 | 79.16 | Inf | 1.6969 | 0.5338 |
| (fear-neutral) - (surprise-neutral) | 43.5 | 33.31 | 79.48 | Inf | 0.4191 | 0.9984 |
| (anger-neutral) - (disgust-neutral) | 43.5 | -87.72 | 78.83 | Inf | -1.1127 | 0.8764 |
| (anger-neutral) - (happiness-neutral) | 43.5 | -687.34 | 79.16 | Inf | -8.6834 | 0.0000 |
| (anger-neutral) - (sadness-neutral) | 43.5 | -145.94 | 78.83 | Inf | -1.8513 | 0.4326 |
| (anger-neutral) - (surprise-neutral) | 43.5 | -246.96 | 78.83 | Inf | -3.1326 | 0.0214 |
| (disgust-neutral) - (happiness-neutral) | 43.5 | -599.62 | 79.03 | Inf | -7.5868 | 0.0000 |
| (disgust-neutral) - (sadness-neutral) | 43.5 | -58.22 | 78.82 | Inf | -0.7387 | 0.9771 |
| (disgust-neutral) - (surprise-neutral) | 43.5 | -159.24 | 78.87 | Inf | -2.0189 | 0.3314 |
| (happiness-neutral) - (sadness-neutral) | 43.5 | 541.40 | 79.03 | Inf | 6.8501 | 0.0000 |
| (happiness-neutral) - (surprise-neutral) | 43.5 | 440.38 | 79.30 | Inf | 5.5532 | 0.0000 |
| (sadness-neutral) - (surprise-neutral) | 43.5 | -101.01 | 78.87 | Inf | -1.2807 | 0.7958 |
| (fear-neutral) - (anger-neutral) | 59.5 | -20.69 | 49.50 | Inf | -0.4180 | 0.9984 |
| (fear-neutral) - (disgust-neutral) | 59.5 | 60.05 | 49.26 | Inf | 1.2190 | 0.8278 |
| (fear-neutral) - (happiness-neutral) | 59.5 | -320.22 | 48.74 | Inf | -6.5692 | 0.0000 |
| (fear-neutral) - (sadness-neutral) | 59.5 | 26.08 | 49.26 | Inf | 0.5293 | 0.9950 |
| (fear-neutral) - (surprise-neutral) | 59.5 | -67.04 | 49.78 | Inf | -1.3467 | 0.7588 |
| (anger-neutral) - (disgust-neutral) | 59.5 | 80.75 | 48.74 | Inf | 1.6565 | 0.5608 |
| (anger-neutral) - (happiness-neutral) | 59.5 | -299.52 | 49.26 | Inf | -6.0800 | 0.0000 |
| (anger-neutral) - (sadness-neutral) | 59.5 | 46.77 | 48.74 | Inf | 0.9595 | 0.9306 |
| (anger-neutral) - (surprise-neutral) | 59.5 | -46.34 | 48.74 | Inf | -0.9507 | 0.9331 |
| (disgust-neutral) - (happiness-neutral) | 59.5 | -380.27 | 49.07 | Inf | -7.7495 | 0.0000 |
| (disgust-neutral) - (sadness-neutral) | 59.5 | -33.98 | 48.72 | Inf | -0.6973 | 0.9823 |
| (disgust-neutral) - (surprise-neutral) | 59.5 | -127.09 | 48.81 | Inf | -2.6037 | 0.0963 |
| (happiness-neutral) - (sadness-neutral) | 59.5 | 346.29 | 49.07 | Inf | 7.0571 | 0.0000 |
| (happiness-neutral) - (surprise-neutral) | 59.5 | 253.18 | 49.50 | Inf | 5.1147 | 0.0000 |
| (sadness-neutral) - (surprise-neutral) | 59.5 | -93.11 | 48.81 | Inf | -1.9077 | 0.3973 |
| (fear-neutral) - (anger-neutral) | 96.5 | -716.65 | 167.83 | Inf | -4.2701 | 0.0003 |
| (fear-neutral) - (disgust-neutral) | 96.5 | -246.33 | 167.76 | Inf | -1.4684 | 0.6846 |
| (fear-neutral) - (happiness-neutral) | 96.5 | -119.35 | 167.61 | Inf | -0.7121 | 0.9806 |
| (fear-neutral) - (sadness-neutral) | 96.5 | -224.24 | 167.76 | Inf | -1.3366 | 0.7646 |
| (fear-neutral) - (surprise-neutral) | 96.5 | -299.08 | 167.91 | Inf | -1.7812 | 0.4780 |
| (anger-neutral) - (disgust-neutral) | 96.5 | 470.32 | 167.61 | Inf | 2.8060 | 0.0565 |
| (anger-neutral) - (happiness-neutral) | 96.5 | 597.30 | 167.76 | Inf | 3.5604 | 0.0050 |
| (anger-neutral) - (sadness-neutral) | 96.5 | 492.42 | 167.61 | Inf | 2.9379 | 0.0388 |
| (anger-neutral) - (surprise-neutral) | 96.5 | 417.57 | 167.61 | Inf | 2.4913 | 0.1265 |
| (disgust-neutral) - (happiness-neutral) | 96.5 | 126.98 | 167.70 | Inf | 0.7572 | 0.9745 |
| (disgust-neutral) - (sadness-neutral) | 96.5 | 22.10 | 167.60 | Inf | 0.1318 | 1.0000 |
| (disgust-neutral) - (surprise-neutral) | 96.5 | -52.75 | 167.63 | Inf | -0.3147 | 0.9996 |
| (happiness-neutral) - (sadness-neutral) | 96.5 | -104.89 | 167.70 | Inf | -0.6254 | 0.9892 |
| (happiness-neutral) - (surprise-neutral) | 96.5 | -179.73 | 167.83 | Inf | -1.0709 | 0.8930 |
| (sadness-neutral) - (surprise-neutral) | 96.5 | -74.84 | 167.63 | Inf | -0.4465 | 0.9978 |

CTQ: total score of the Childhood Trauma Questionnaire; Estimate: difference of estimated marginal means (in milliseconds) between the contrasted conditions; SE: standard error; df: degrees of freedom; t: t statistic.

**Figure S1: Fixation durations on the emotional and neutral facial expressions for fearful-, angry-, disgusted-, happy-, sad-, and surprised-neutral face pairs. Error bars denote standard error.**

*
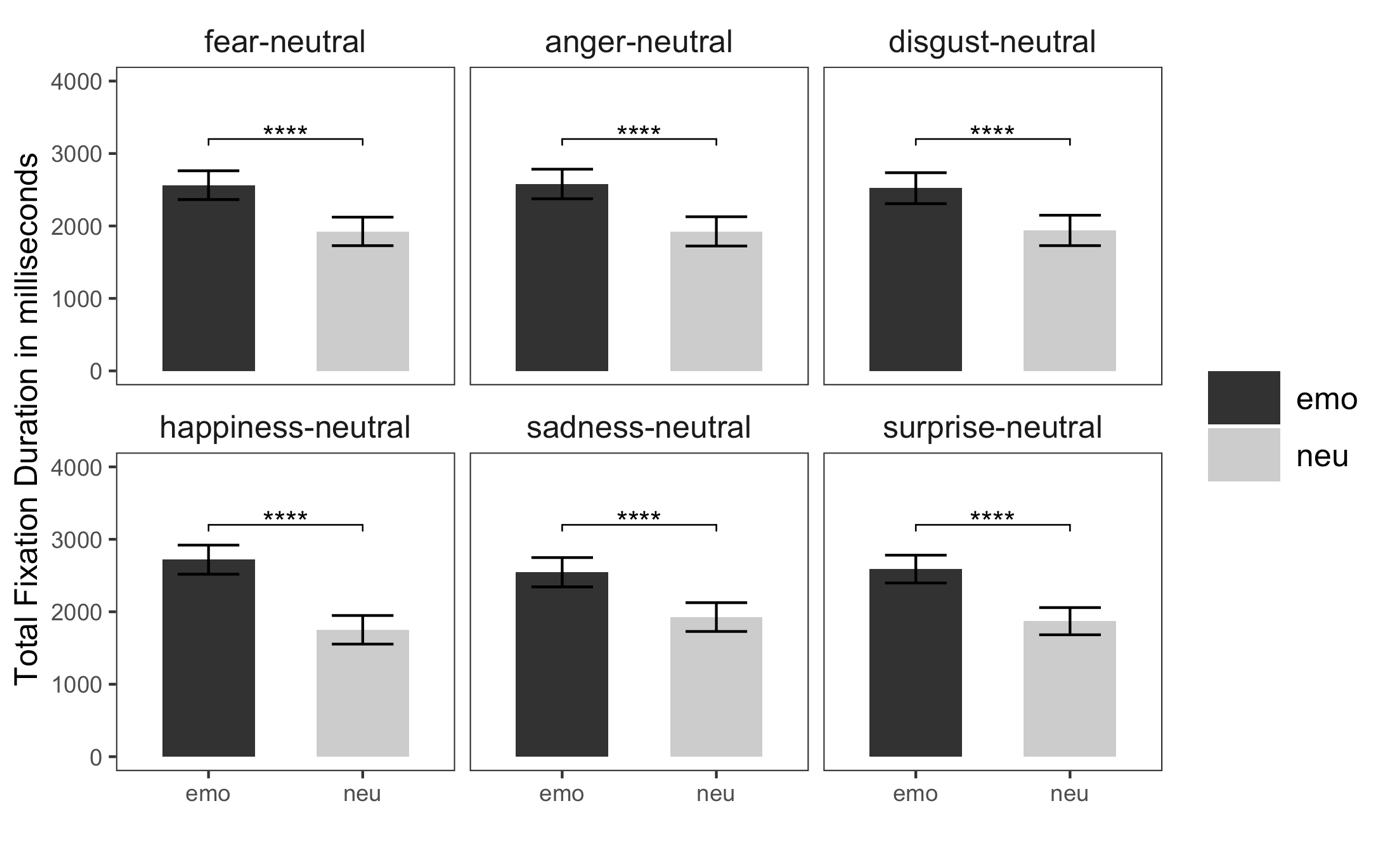
*

****** Significant difference at *p* < .0001.**
